# Pervasiveness of HLA allele-specific expression loss across tumor types

**DOI:** 10.1186/s13073-023-01154-x

**Published:** 2023-02-09

**Authors:** Ioan Filip, Anqi Wang, Oleksandr Kravets, Rose Orenbuch, Junfei Zhao, Tomin E. Perea-Chamblee, Gulam A. Manji, Evangelina López de Maturana, Núria Malats, Kenneth P. Olive, Raul Rabadan

**Affiliations:** 1 Program for Mathematical Genomics, Department of Systems Biology, Columbia University, New York, NY USA; 2 Systems Biology, Harvard Medical School, Boston, MA USA; 3 Department of Medicine, Division of Hematology and Oncology, Columbia University, New York, NY USA; 4 Genetic and Molecular Epidemiology Group, Spanish National Cancer Research Centre (CNIO), and CIBERONC, Madrid, Spain; 5 Department of Medicine, Division of Digestive and Liver Diseases, Columbia University, New York, NY USA; 6 Department of Biomedical Informatics, Columbia University, New York, NY USA

**Keywords:** HLA, Allele-specific expression, Loss of heterogeneity, Pan-cancer analysis, Pancreatic cancer, Immunotherapy

## Abstract

**Background:**

Efficient presentation of mutant peptide fragments by the human leukocyte antigen class I (HLA-I) genes is necessary for immune-mediated killing of cancer cells. According to recent reports, patient HLA-I genotypes can impact the efficacy of cancer immunotherapy, and the somatic loss of HLA-I heterozygosity has been established as a factor in immune evasion. While global deregulated expression of HLA-I has also been reported in different tumor types, the role of HLA-I allele-specific expression loss — that is, the preferential RNA expression loss of specific HLA-I alleles — has not been fully characterized in cancer.

**Methods:**

Here, we use RNA and whole-exome sequencing data to quantify HLA-I allele-specific expression (ASE) in cancer using our novel method *arcasHLA-quant*.

**Results:**

We show that HLA-I ASE loss in at least one of the three HLA-I genes is a pervasive phenomenon across TCGA tumor types. In pancreatic adenocarcinoma, tumor-specific HLA-I ASE loss is associated with decreased overall survival specifically in the basal-like subtype, a finding that we validated in an independent cohort through laser-capture microdissection. Additionally, we show that HLA-I ASE loss is associated with poor immunotherapy outcomes in metastatic melanoma through retrospective analyses.

**Conclusions:**

Together, our results highlight the prevalence of HLA-I ASE loss and provide initial evidence of its clinical significance in cancer prognosis and immunotherapy treatment.

**Supplementary Information:**

The online version contains supplementary material available at 10.1186/s13073-023-01154-x.

## Background

Somatic mutations and chromosomal instability drive carcinogenesis and progression of cancer. Mutant peptide fragments derived from aberrant proteins can trigger a cytotoxic T-cell response through recognition of neoantigens that differ sufficiently from the normal host peptides [1]. As HLA-I is necessary for neoantigen presentation in cancer cells, disruptions in HLA-I expression can have major implications on immune evasion. Meta-analyses of human cancers indicate abnormal global HLA-I expression in particular for non-small cell lung cancer, breast carcinoma, head-neck squamous cell carcinoma, melanoma, as well as bladder, pancreas, and prostate tumors, in up to 90% of primary samples [2–5].

Although down-regulation of HLA-I can allow tumor cells to escape immune detection by cytotoxic T-cells, a complete loss of HLA-I makes cells vulnerable to natural killer (NK) antitumor activity as they are no longer able to present self-antigens on the cell surface [6]. Indeed, down-regulation of HLA-I is associated with worse prognosis [3, 7], but it is also associated with a decreased metastatic potential [8]. The tumor microenvironment, therefore, plays a critical role in immune escape [3], and it has been suggested that decreased expression of HLA-I, but not complete loss, can allow tumors to escape from both T-cell and NK surveillance [9].

The loss of HLA-I germline heterozygosity (LOH), through either partial or complete loss of chromosome 6 or a focal deletion of the HLA locus, is a common molecular mechanism that may drive abnormal HLA-I expression [5]. LOH, traditionally assessed through analysis of microsatellite markers, is frequently observed in many tumor types, including head-neck [10] and pancreatic cancer [11]. Haplotype-specific copy number inference through computational approaches has enabled LOH assessment from standard next-generation DNA sequencing, showing that LOH occurs in 40% of non-small-cell lung cancers [12].

However, the LOH of HLA-I genes, which occurs at the genomic level, is not identical to HLA allele-specific mRNA expression (ASE) loss, which is measured at the transcriptomic level. While a few works have recently reported HLA-I ASE [13, 14], even at single-cell resolution [15], there is currently no gold standard for measuring HLA-I ASE from RNA-seq data. Furthermore, our understanding of the clinical significance of HLA-I imbalance at the level of expression (HLA-I ASE loss) remains incomplete across cancer types.

To systematically characterize HLA-I ASE loss across tumor types, we developed an allele-specific quantification method, namely *arcasHLA-quant*, which builds upon our previously established high-resolution HLA genotyping protocols based on RNA-seq data [16, 17]. In light of ubiquitous HLA-I aberrant expression in cancer, we hypothesized that HLA-I ASE loss (specifically, loss of either HLA-A, HLA-B, or HLA-C) may constitute a universal immune escape mechanism with significant clinical impact, particularly in the context of immunotherapy. Using *arcasHLA-quant*, we first quantified HLA-I ASE in cancer tissue for ~ 9 k individuals across thirty-two TCGA molecular tumor subtypes [18]. Interestingly, we observed many cases with HLA-I ASE loss did not possess DNA-level HLA-I LOH, suggesting the existence of additional factors which may impact expression imbalance in tumor samples. While our analysis did not identify any universal effect of HLA-I ASE loss on prognosis, in the basal-like subtype of pancreatic ductal carcinoma HLA-I ASE loss was associated with worse overall survival. We also found that HLA-I ASE loss was associated with a poor outcome to anti-PD-1 immunotherapy treatment for metastatic melanoma.

## Methods

### Cohort descriptions

#### TCGA

We have included 9000 tumors from the Cancer Genome Atlas (TCGA) [18] across 32 molecular subtypes where whole exome sequencing (WES) samples were available from the tumor and from normal tissue, in addition to matched RNA-seq derived from the same tumor sample. The TCGA-LAML dataset was not included in our study because of the lack of primary tumor samples. We also eliminated TCGA cases from the TCGA-PAAD dataset that were not classified as pancreatic ductal adenocarcinoma using the criteria laid out in [19]. We dub the resulting subset as “TCGA-PDAC” henceforward. In order to reduce the bias caused by low purity, we excluded cases with ultra-low purity estimate (sequenza-inferred purity below 10%; see the “Tumor purity and ploidy inference” section below). After the filter, 8,182 cases remained and the HLA-I ASE loss ratios were calculated based on these TCGA cases. For survival analysis, we excluded cases without age at diagnosis information and cases without overall survival data. We also excluded DLBC, PCPG, TCTG and THYM from survival analysis per the recommendation in [20] given that the sample size or the number of events regarding overall survival is too small in these TCGA cohorts. We further excluded THCA in survival analysis because of the low percentage of cases with tumor purity > 0.1 (as assessed with our computational pipeline*;* see Tumor purity and ploidy inference).

#### CUMC cohort

We analyzed a previously published cohort at Columbia University [21] (denoted as CUMC, *n* = 192) comprised of epithelial samples (CUMC-E, *n* = 96) and stroma samples (CUMC-S, *n* = 96) that were cleanly delineated through laser-capture microdissection and subsequently processed and sequenced separately. The CUMC cohort consists of patients who underwent surgery at the Columbia Pancreas Center. Specimens were harvested and frozen intraoperatively by the Columbia University Tumor Bank in collaboration with the Columbia Pancreas Center. All *n* = 96 cases reported here were diagnosed as PDAC and had complete RNA-seq data as well as overall survival information available. The vast majority of samples (94%) were stage 2A or 2B. We divided the CUMC-E samples into basal-like and classical subtypes using the method reported in [22].

#### Metastatic melanoma cohort

**Fig. 1:**
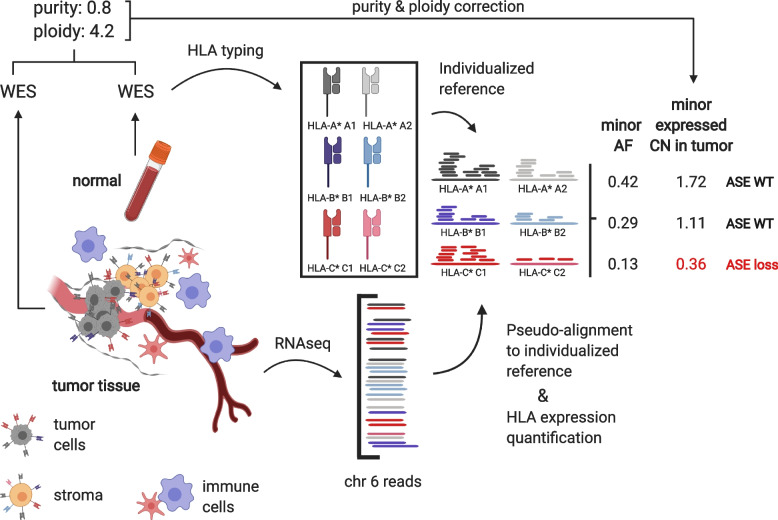
Workflow for quantification of HLA-I allele-specific expression loss using *arcasHLA-quant*. The pipeline takes RNA-seq data from tumor, and WES data from tumor and normal samples. The major steps include (1) HLA-I genotyping; (2) HLA-I gene expression quantification using *arcasHLA-quant* and allele frequency calculation; (3) tumor purity and ploidy estimation; and (4) adjustment of allele frequencies for HLA-I ASE inference

For the analysis of HLA-I ASE loss in the context of immunotherapy, we included a retrospective study of pre- and on-treatment samples in metastatic melanoma [23]. In total, 85 patients were accrued across multiple study arms and institutions in order to evaluate the pharmacodynamic activity of Nivolumab. Patients received Nivolumab every 2 weeks until progression (or for a maximum of 2 years). They underwent biopsy twice: once before the treatment start (1–7 days before the first dose; referred to as the pre-treatment sample in our paper), and a second time on cycle 1, day 29 (between 23 and 29 days), collected at the same site (referred to as the on-treatment sample). In all, we identified *n* = 75 cases (*n* = 46 pre-treatment and *n* = 29 on-treatment) with paired DNA and RNA samples as required for our pipeline (see Fig. 1). For all analyses with the metastatic melanoma cohort, we excluded cases with ultra-low purity (sequenza-inferred purity < 0.1). After filtering, there were *n* = 41 pre- and *n* = 25 on-treatment cases remaining.

### HLA-I allele-specific expression (ASE) quantification with *arcasHLA-quant*

We propose a novel pipeline (Fig. 1) to quantify the loss of HLA allele-specific expression in HLA-A, HLA-B, and HLA-C. The input consists of RNA-seq data from tumor tissue and whole exome sequencing (WES) data from tumor tissue and from the paired normal sample. The output is the tumor-specific expressed copy number of each allele in HLA genes. These inferred tumor-specific expressed copy numbers are the basis of identifying whether HLA-I ASE loss occurs or not in the input tumor sample. Our pipeline contains the following key steps (Fig. 1): (1) identification of the genotype of every HLA gene based on WES data from the normal sample; (2) quantification of allele-specific expression of every HLA gene from RNA-seq data using *arcasHLA-quant*, and calculation of allele frequency; (3) estimation of tumor purity and ploidy by comparing the WES data from tumor and normal samples; (4) adjustment of allele frequency by taking into account the estimated tumor purity and ploidy, which we denote here as “expressed copy number.” Finally, we define tumor HLA ASE loss in the cases with detectably high HLA expression imbalance in minor-major allele pairs for any of the HLA-I genes (namely in HLA-A, HLA-B, or HLA-C).

In step (1), when normal WES samples are not available, *arcasHLA-quant* can also quantify allele-specific expression by taking the genotype that is determined using *arcasHLA* [16] from RNA-seq data. In step (2), similar to existing approaches [13], the *arcasHLA-quant* method first builds a customized transcriptome reference by replacing the default HLA transcripts from the human chromosome 6 reference (GRCh 38) with patient-specific HLA-I allelic cDNA references obtained from the IMGT/HLA database [24]. Subsequently, reads from input BAM files are extracted as in *arcasHLA*, and allele-specific expression quantification is performed using Kallisto [25]. This approach extends the workflow and applicability of *arcasHLA*; importantly, the same pipeline for extracting reads from input samples and constructing graph-based references for pseudo-mapping — which give *arcasHLA* high-resolution accuracy in genotyping HLA class I and class II genes from RNA-seq — are used for *arcasHLA-quant*. *arcasHLA-quant* is developed in Python and can be run as a command-line instruction set or in a virtual environment. It has been incorporated into *arcasHLA* and it is publicly available: https://github.com/RabadanLab/arcasHLA [26].

### HLA-I genotyping and HLA supertypes

For all the TCGA cohorts in this study, high-resolution HLA class I genotyping was performed with Polysolver [27] from normal WES samples. We noticed that the Polysolver HLA-I genotyping results for a part of the cases were previously available [18]. All HLA supertypes [28] were annotated in the TCGA cohort for each subject and included as binary predictor variables in the multivariate Cox regressions. For the CUMC cohort, high-resolution HLA-I genotyping was performed from RNA-seq using *arcasHLA* [16]. Only the stromal compartment was used to infer patient HLA genotypes. For the metastatic melanoma cohort, the HLA-I genotypes were previously available [29].

### Tumor purity and ploidy inference

We used the sequenza [30] algorithm with default parameters to obtain purity and ploidy estimates for all the TCGA samples, and likewise for the samples from the metastatic melanoma cohort. Among all the solutions proposed by the model, we selected the purity-ploidy pair with the highest posterior probability. Owing to the absence of DNA sequencing data, we assumed that the laser-capture microdissected CUMC-E and CUMC-S samples had 100% purity, and ploidy equal to 2.0 for the purpose of calculating ASE loss in the CUMC cohort.

### Assessment of HLA-I allele-specific expression loss (HLA-I ASE loss)

In order to determine the status of HLA-I ASE loss in the tumor component of bulk RNA-seq, we incorporated the following two pieces of information: 1) tumor purity and ploidy inferred from paired tumor and normal samples; and 2) HLA-I ASE inferred from RNA-seq using *arcasHLA-quant*. Similar to a previously published criterion for somatic LOH, LOHHLA [12], we determined a purity- and ploidy-adjusted tumor-expressed copy number for each HLA-I allele.

Denote ${AF}_{i}$ ($i=1, 2$) as the allelic frequency, which is namely the ratio of reads attributed to each allele over the total read count for the corresponding HLA gene in the bulk sample. Denote $\rho$ as the tumor purity and $\psi$ as the overall tumor ploidy, which are obtained from sequenza [30]. Our aim is to infer ${exp}_{{CN}_{i}}$ ($i=1, 2$), which is the relative expression of an allele in a tumor cell over a normal cell. We term ${exp}_{{CN}_{i}}$ as the “expressed copy number” hereafter. Assume that the expressed copy numbers for both alleles are equal to 1 in the normal cells, then the expressed copy number for each allele in the sample is

$$\rho\cdot{exp}_{{CN}_i}+\left(1-\rho\right),$$

which is the weighted sum of those in the tumor cells and normal cells. The allele frequency, which we could observe based on the read count, can also be defined as

$${AF}_i=\frac{\rho\cdot{exp}_{{CN}_i}+\left(1-\rho\right)}{\rho\left({exp}_{{CN}_1}+{exp}_{{CN}_2}\right)+2\left(1-\rho\right)}.$$

We normalize the sum of the expressed copy numbers of the two alleles as the ploidy,

$${exp}_{{CN}_{1}}+{exp}_{{CN}_{2}}=\psi ,$$

and the expressed copy number of each allele in the tumor is

1 $${exp}_{{CN}_{i}}=\frac{1}{\rho }\left(2{AF}_{i}\left(1+\rho \frac{\psi -2}{2}\right)-\left(1-\rho \right)\right).$$

As in LOHHLA, we defined ASE loss as the occurrence of a minor allele exp_CN_ below 0.5 in at least one HLA-I gene (HLA-A, HLA-B, or HLA-C). We note that the above formula yields an expressed copy number (exp_CN_) of 1 for both minor and major alleles in the case of a heterozygous HLA-I gene with perfectly balanced allelic expression levels (i.e. with $AF = 0.5$), and 100% tumor purity ($\rho =1.0$) and normal ploidy ($\psi = 2.0$). It should be noticed that the cutoff 0.5 was selected as an analogy to the DNA case. While our validation shows it yielded a specificity of 97.9% (see Results), the thresholding could still benefit from advanced work in the future due to the caveat that loss in DNA and RNA may not be equivalent.

### HLA-I ASE loss and nonsense or missense mutations

Nonsense mutations on HLA-I genes for TCGA cases were collected from [31]. There were in total 58 events of “(case, HLA-I gene)” combination being identified. For each of the HLA-I gene, we performed a Fisher’s exact test to investigate if HLA-I ASE loss was enriched in cases with nonsense mutations. Only the cases that had heterozygous genotype on the associated HLA-I gene were included in the test. Then we performed Fisher’s exact test to check the enrichment of HLA-I ASE loss event in any of the HLA-I genes. The cases that had heterozygous genotype for at least one of the HLA-I genes were involved. The same analysis was also carried out for HLA-I missense mutations, and nonsense/missense B2M mutations [27].

### Assessment of somatic loss of HLA-I haplotypes

We used LOHHLA [12] to infer HLA-I allele-specific copy number variation and determine somatic LOH at the level of DNA, from input tumor and normal paired WES samples. We set the minimum coverage threshold at 5 and used the default configuration for all other parameters. In this study, we focused on somatic LOH cases that also exhibited HLA-I ASE loss*.* We used the criteria for LOH-positive as indicated by LOHHLA, namely: allelic copy number (CN) < 0.5 and *p*-value < 0.05. Cases that resulted in LOHHLA errors were excluded from the comparison with *arcasHLA-quant* ASE loss*.*

### In silico decomposition into immune cell subtypes

We used CIBERSORTx [32] LM22 signature matrix containing twenty-two functionally defined human immune-cell subtypes to quantify the immune cell infiltration in the tumor RNA-seq samples. We used the CIBERSORTx support-vector machine approach with default parameters for each sample in TCGA. However, since the method produces a weight decomposition of each bulk sample into fractional contributions from each immune subtype that sum to 1, this method is not entirely adequate for separating tumor cell signatures from immune cell signatures since it does not include a tumor component in the final decomposition. Owing to a lack of normal tissue expression signatures for each corresponding TCGA cohort in our study, for each tumor bulk sample, we corrected every immune cell proportions by only retaining the immune cell subtypes reported by CIBERSORTx that had a fractional contribution exceeding 10%. Subsequently, we defined the following immune features for Cox regression analyses by adding the latter corrected LM22 subtype fractional parts according to their corresponding immune lineage category: CD4 + T cells, CD8 + T cells, B cells, Macrophages, NK cells, and other Macrophages. For example, the B-cell category was defined as the sum of corrected proportions for the following LM22 subtypes: “B cells memory”, “B cells naïve” and “Plasma cells”.

### Computational identification of neopeptides

We used the pVAC-seq pipeline [33] with the MHCflurry and MHCpan binding strength predictors to identify neoantigens [34]. As required, we used the variant effect predictor from Ensembl to annotate variants for downstream processing by pVAC-Seq [35]. For each single-residue missense alteration, HLA-I allele-specific binding affinities were predicted for all the wild-type and mutant peptide fragments of varying lengths (from 8 to 11 amino acids). The mutant peptide with the strongest binding affinity was kept for downstream analysis. The total potentially immunogenic neoantigen count was determined for each individual as the number of predicted mutant epitopes with a median IC50 score below 500. This feature, called “total neoantigen count”, was subsequently included in the Cox model of overall survival. Two additional neoantigen counts that were adjusted for HLA-I ASE loss status, “neoantigen count: affinity to lost allele” and “neoantigen count: affinity to kept allele”, were also included in the model. They were defined as the counts of identified neoantigens that had strongest binding affinity to the lost/kept HLA allele.

## Results

### Quantifying HLA allele-specific expression using *arcasHLA-quant*

As a consistency check, we verified that gene-specific quantification levels obtained with *arcasHLA-quant* by summing minor and major allele expression for each HLA-I gene (HLA-A, HLA-B, and HLA-C) were consistent with expression levels as inferred through alternate methods [36] available on the TCGA portal (Pearson’s correlation coefficients in the range 0.94–0.96, *p* < 10^−16^ across tumor subtypes, Additional file 1: Fig. S1). *arcasHLA-quant* also yielded consistent results with HLApers [13] in terms of HLA-I gene quantification (Additional file 1: Fig. S2) and minor allele frequency inference (Additional file 1: Fig. S3), which was calculated as the number of reads supporting the allele with fewer reads over the total number of reads per HLA-I gene locus.

In order to evaluate the accuracy of HLA-I ASE loss calling, we took the TCGA normal cases as a negative control and the TCGA tumor cases with heterozygous loss of HLA-I genes at the DNA level as a positive control. We assumed that allelic loss at DNA level in the tumor would also be detectable at the level of mRNA expression. The positive control cases were manually validated LOHHLA calls with high confidence. Among 382 positive control cases, 324 were identified to have HLA-I ASE loss, which indicates an overall sensitivity of 84.8%. The positive control samples covered a wide range of tumor purity values (from 10% to > 90%). Among the 703 negative control cases which we took as a negative control, 15 were called as HLA-I ASE loss, indicating a specificity of 97.9% (688/703). The area under the receiver operating curve (AUC) is 0.921 (Additional file 1: Fig. S4).

### HLA-I allele-specific expression loss is pervasive across tumor types

**Fig. 2:**
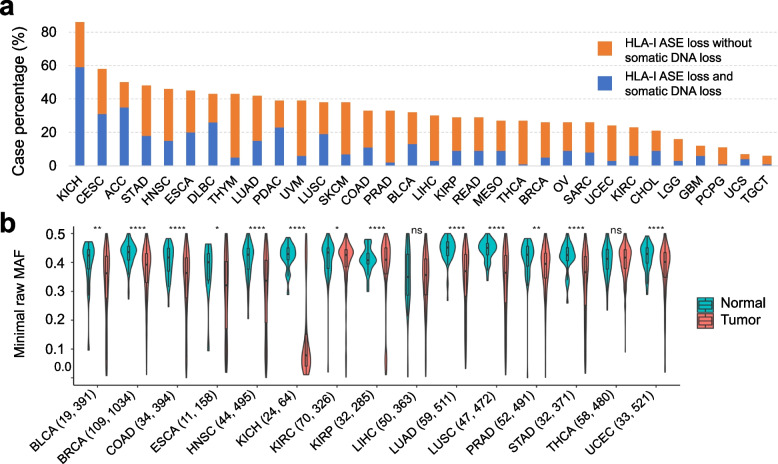
Pervasiveness of HLA-I allele-specific expression loss. **a** Proportions of HLA-I ASE loss across TCGA subtypes (orange bars) as inferred using *arcasHLA-quant*. Blue (orange) bars represent proportions of cases where expression loss is (not) accompanied by somatic DNA loss, as inferred by LOHHLA on WES data. **b** HLA-I ASE comparison between tumor and normal cases in TCGA cohorts. HLA-I ASE is captured by the minimal raw minor allele frequency among the three HLA-I genes (minimal raw MAF). Numbers in the parentheses indicate the normal and tumor case numbers respectively. Only the TCGA cohorts with more than 10 normal cases are shown. Significance labels: “ns” or nothing labeled: *p* > 0.05; “*”: *p* < 0.05; “**”: *p* < 0.01; “***”: *p* < 0.001; “****”: *p* < 0.0001

We first determined that HLA-I ASE loss is pervasive across TCGA tumor types (Fig. 2a): ASE loss was detected in every tumor type analyzed, most prominently in kidney chromophobe (KICH), with a frequency of 86%, followed by another eight tumor types that exhibited frequencies greater than 40%, including cervical squamous cell carcinoma (CESC, 58%), adrenocortical carcinoma (ACC, 50%), stomach adenocarcinoma (STAD, 48%), head and neck squamous cell carcinoma (HNSC, 46%), esophageal adenocarcinoma (ESCA, 45%), diffuse large B-cell lymphoma (DLBC, 43%), thymoma (THYM, 43%) and lung adenocarcinoma (LUAD, 42%). Five tumor types showed a markedly lower incidence of HLA-I ASE loss rates, including lower-grade glioma (LGG, 16%), glioblastoma (GBM, 12%), pheochromocytoma and paraganglioma (PCPG, 11%), uterine carcinosarcoma (UCS, 7%) and testicular germ cell tumors (TGCT, 6%). In order to limit ASE loss calling errors due to extremely low purity levels, we filtered out TCGA samples with purity below 0.1 (Additional file 1: Fig. S5). Overall, HLA-I ASE loss was attributable to HLA-A in 27% of cases, to HLA-B in 22% and to HLA-C in 25% of cases, with loss at all three genes occurring at a rate of 13% (Additional file 1: Fig. S6).

We then investigated whether HLA-I ASE loss was accompanied by somatic DNA lesions (e.g., chromosomal or focal deletions) at the corresponding HLA-I locus. Using LOHHLA [12], we found that only a fraction of ASE losses showed evidence of DNA haplotype loss (Fig. 2a). The maximal proportion of DNA-to-expression-only loss was found in ACC (70%), while THCA had the smallest such proportion (under 4%). Our results suggest that a major proportion of HLA-I ASE loss in cancer may occur through epigenetic or other expression regulatory mechanisms instead of somatic DNA lesions.

We further compared HLA-I expression between the TCGA tumor samples and the paired normal samples, where available. We found that TCGA tumor samples had significantly lower minor allele frequency in most cohorts (Fig. 2b). We posited that the extensive allelic imbalance observed in many tumors was primarily due to the tumor component in each of the bulk samples analyzed.

We also studied the association between HLA-I ASE loss and microsatellite instability (MSI) in TCGA cohorts [37]. Due to the low reported frequency of MSI, only three cohorts (UCEC, COAD, and STAD) had more than 20 MSI cases (Additional file 2: Table S1). We found that the MSI cases consistently had higher HLA-I ASE loss frequency compared with non-MSI cases in the three cohorts: 53% vs 30% in COAD (odds ratio, OR = 2.65, *p* = 1.8 $\times$ 10^−4^; Fisher’s exact test); 65% vs 52% in STAD (OR = 1.69, *p* = 0.075; Fisher’s exact test); and 38% vs 25% in UCEC (OR = 1.75, *p* = 5.5 $\times$ 10^−3^; Fisher’s exact test).

Disrupted HLA-I expression can also result from the accumulation of somatic mutations in HLA-I [5]. However, somatic mutations in HLA-I are relatively infrequent in TCGA, varying from below 1% incidence in BRCA and GBM, to around 5% in BLCA, LUAD, and SKCM, and up to 10% in HNSC [7, 27]. Using a comprehensive list of HLA-I mutations in TCGA [31], we identified 58 nonsense HLA-I mutation events. Nonsense mutations in HLA-I genes are expected to lead to a severe imbalance of mRNA from the mutant allele, as compared to the wild-type allele, through nonsense-mediated decay. Consistent with this assumption, we found an enrichment of HLA-I ASE loss in the cases with nonsense HLA-I mutations (OR = 2.34, *p* = 2.8 $\times$ 10^−3^; Fisher’s exact test; Additional file 2: Table S2a). Several factors may limit such an analysis: (a) the nonsense mutations may not be clonal, but only sub-clonal; (b) there may be issues with coverage in both DNA and RNA data; and (c) nonsense mutations result in premature termination of translation and it is not a priori clear that mRNAs are degraded or eliminated at such a rate so as to be detectable in RNA-seq. Despite these obstacles in detecting HLA-I ASE loss from nonsense mutations, we were still able to observe a significant enrichment (Additional file 2: Table S2a), further reinforcing the validity of our approach for assessing HLA-I ASE loss. We performed the same analyses on HLA-I missense mutations [27]. Interestingly, HLA-I ASE loss was also significantly enriched in the cases with HLA-B missense mutations (OR = 2.46, *p* = 5.4 $\times$ 10^−3^; Fisher’s exact test; Additional file 2: Table S2b). The enrichment maintained significance when looking solely at the HLA-B missense mutations corresponding to contact residues (OR = 3.49, *p* = 0.012; Fisher’s exact test; Additional file 2: Table S2c), which was defined by [27] as the mutations in HLA positions that are in actual physical contact with the peptide. We also found that HLA-I ASE loss had significant enrichment in the cases with B2M missense mutations (OR = 2.35, *p* = 0.018; Fisher’s exact test; Additional file 2: Table S2d).

We assessed whether the presence of HLA-I ASE loss resulted in any survival time impact across tumor types. Specifically, a multivariate Cox regression stratified by tumor type was conducted as a pan-cancer analysis to investigate the clinical significance of HLA-I ASE loss. A total of 27 tumor sample features were taken as covariates, including age at diagnosis, tumor purity and ploidy estimates, and several immune-related and microenvironmental features (Additional file 1: Fig. S7). While we noticed that age at diagnosis, tumor ploidy, and macrophages were significantly associated with shorter overall survival, HLA-I ASE loss did not show a significant pan-cancer effect in this model (Additional file 1: Fig. S7). We also conducted univariate cox-regression models for every TCGA cohort but found no significant cohort-wide association after correcting for multiple hypothesis testing (Additional file 1: Fig. S8). However, HLA-I ASE loss showed a trend towards worse survival in KIRP (*n* = 264 cases; HR = 1.38, nominal *p* = 0.021; Additional file 1: Fig. S8a). The same trend was observed when the predicted neoantigen count was added to the regression model (HR = 1.43, *p* = 7.11 $\times$ 10^−3^; Additional file 1: Fig. S8b). Additionally, HLA-I ASE loss showed a trend towards poorer prognosis in PDAC when the predicted neoantigen count was considered (*n* = 130 cases; HR = 1.24, nominal *p* = 0.058; Additional file 1: Fig. S8b). The difference in prognosis between cases with HLA-I ASE loss and those without HLA-I ASE loss was sharper among the patients with overall survival shorter than 24 months (Additional file 1: Fig. S9). Finally, among patients with heterozygous genotypes at all three HLA-I loci, there was a marked difference in prognosis as well (LR = 6.28; *p* = 0.01; log-rank test; Additional file 1: Fig. S10). Next, we evaluated these association trends with survival, observed at cohort level, by further dissecting our analysis into molecular subtypes and tumor stage.

### HLA-I allele-specific expression loss is associated with decreased overall survival in the basal-like PDAC subtype

**Fig. 3:**
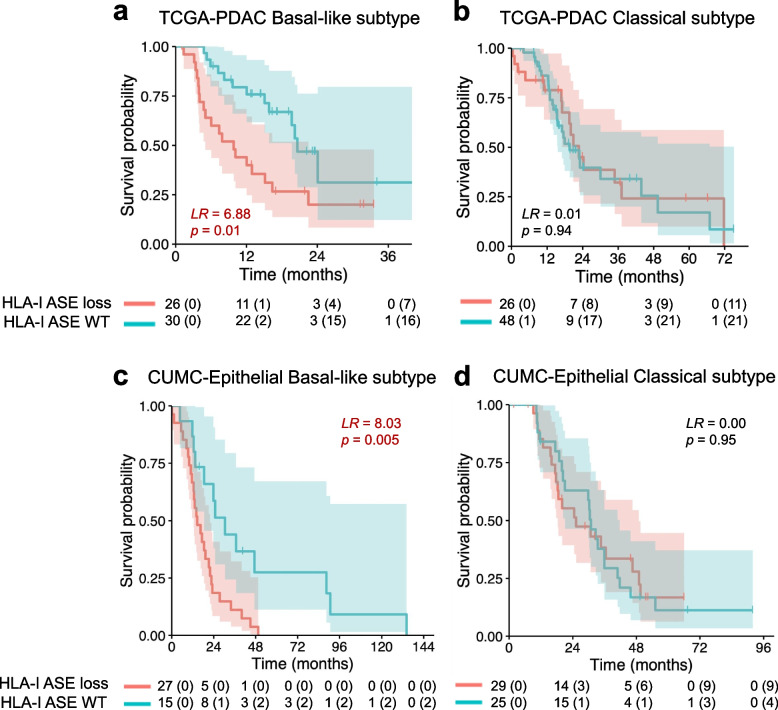
HLA-I allele-specific expression loss in basal-like and classical transcriptional subtypes of pancreatic adenocarcinoma. Survival curves in **a** TCGA-PDAC basal-like subtype; **b** TCGA-PDAC classical subtype; **c** CUMC-Epithelial PDAC basal-like subtype; and **d** CUMC-Epithelial PDAC classical subtype. Cases with ultra-low purity (< 0.1) were filtered out. Log-rank scores and *p*-values are indicated

We interrogated the potential associations between HLA-I ASE loss in pancreatic cancer and the well-characterized classical/basal-like transcriptional subtypes [22, 38–40]. We found that ASE loss was present in both PDAC subtypes with some enrichment in the basal-like tumors (OR = 1.59, *p* = 0.21; Fisher’s exact test) which was not significant. Moreover, detection of HLA-I ASE loss in the basal-like subtype, but not in the classical one, was associated with worse survival (LR = 6.88, *p* = 0.01; log-rank test; Fig. 3a, b), suggesting the existence of a basal-like subcategory of PDAC characterized by HLA-I ASE loss and poorer prognosis. This result was basically consistent with alternate definitions of transcriptional subtypes in PDAC (Additional file 1: Fig. S11-12). We also noticed that HLA-I ASE detected at AJCC stage 2B had a significant association with shorter survival (LR = 5.19, *p* = 0.02; log-rank test; Additional file 1: Fig. S13). Additionally, HLA-I ASE loss was detected in earlier stages too, although without a noticeable effect (Additional file 1: Fig. S13).

Next, we validated widespread HLA-I ASE loss in an independent cohort of 96 laser-capture micro-dissected pancreatic ductal adenocarcinoma samples [21] where RNA-seq was performed separately on cleanly delineated epithelial and stroma compartments (CUMC cohort: CUMC-E for epithelial samples and CUMC-S for the stroma; see Cohort descriptions in Methods). Indeed, HLA-I ASE loss was strongly associated with the tumor epithelial compartment (OR = 3.95, *p* = 9.7 $\times$ 10^−6^; Fisher’s exact test), which further supports our hypothesis that HLA-I ASE loss occurs in the cancer cells. Consistent with our previous TCGA analysis, HLA-I ASE loss was linked with shorter survival when detected in CUMC-E (LR = 3.97, *p* = 0.05; log-rank test; Additional file 1: Fig. S14). At the transcriptional subtype level in CUMC-E, HLA-I ASE loss was significantly associated with shorter survival in the basal-like tumors (LR = 8.03, *p* = 0.005; log-rank test; Fig. 3c), while no significant trend was observed in the classical subtype (LR = 0.00, *p* = 0.95; log-rank test; Fig. 3d). Combining the power of both the TCGA-PDAC and the CUMC-E basal-like cohorts strengthened the association with poor survival for HLA-I ASE loss cases (*p* = 1.02 $\times$ 10^−3^, Fisher’s combined probability test). Altogether, our findings indicate that HLA-I ASE loss is a prognostic marker of shorter overall survival in the basal-like subtype of pancreatic ductal adenocarcinoma.

We performed a similar analysis in TCGA-KIRP stratified by subtypes (P-e.1a, P-e.1b, P-e.2, and P.CIMP-e) and stages (I, II, III, and IV), without identifying any new subtype associations (Additional file 1: Fig. S15).

### HLA-I allele-specific expression loss is associated with poor outcomes in anti-PD-1 immunotherapy-treated metastatic melanomas

**Fig. 4:**
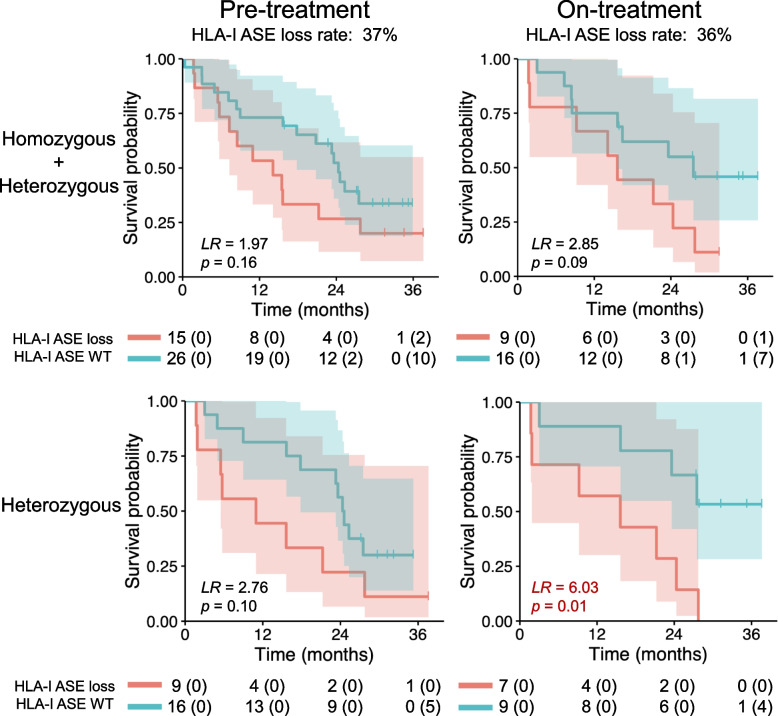
HLA-I allele-specific expression loss and poor outcomes to anti-PD-1 immunotherapy treatment for metastatic melanoma. Extensive HLA-I ASE loss was found in melanoma cohort pre- and on-treatment with Nivolumab. Heterozygous cases are those with heterozygous genotypes for all the three HLA-I genes

Finally, we hypothesized that HLA-I ASE loss may be a factor in the efficacy of immune checkpoint blockade immunotherapies. We revisited a previously published metastatic melanoma cohort [23] with pre- (*n* = 46) and on- (*n* = 29) Nivolumab therapy samples (see the “Cohort descriptions” section) and inferred HLA-I ASE loss as described before. Excluding samples with ultra-low tumor purity (below 0.1), we found ASE loss in both pre- and on-therapy samples with frequencies around 37% (Fig. 4). Furthermore, ASE loss was associated with worse overall survival regardless of whether it was assessed before or during therapy. The group with on-therapy ASE loss showed a slightly greater effect on prognosis (LR = 2.85, *p* = 0.09; log-rank test; Fig. 4).

It has been previously reported that HLA class I homozygosity can reduce overall survival with immune checkpoint blockade [29] (Additional file 1: Fig. S16). As such, we also analyzed the impact of HLA-I ASE loss separately for individuals heterozygous at all three HLA-I genes. For these individuals, HLA-I ASE loss was associated with significantly worse prognoses (LR = 6.03, *p* = 0.01; log-rank test; Fig. 4) when expression loss occurred on-therapy (1 month after the start of therapy [23]). To a large extent, the association with decreased survival for heterozygous individuals was observed even before therapy, although these results are not as conclusive (LR = 2.76, *p* = 0.10; log-rank test; Fig. 4). The survival impact for the fully heterozygous cohort was also observed when we took neoantigen predictions into account (LR = 6.91, *p* = 0.01; log-rank test; Additional file 1: Fig. S17). Results with the full cohort (including cases with ultra-low tumor purity) showed the same trend towards worse prognosis, particularly for heterozygous individuals with on-treatment ASE loss (LR = 4.06, *p* = 0.04; log-rank test; Additional file 1: Fig. S18). Interestingly, among the heterozygous individuals with on-treatment samples and RECIST v1.1 [41]-defined response (*n* = 17), there were only 3 responders (complete or partial), none of which exhibited HLA-I ASE loss (OR = inf., *p* = 0.21; Fisher’s exact test). Among pre-treatment samples, HLA-I ASE loss resulted in slightly lower odds of responding to subsequent treatment (OR = 0.67, n. s.; Fisher’s exact test). In addition, survival associations were not explained by factors such as sample purity (Additional file 1: Fig. S19). In conclusion, our results highlight a potential significant clinical impact of HLA-I ASE loss on the efficacy of anti-PD-1 immunotherapy in metastatic melanoma.

## Discussion

HLA-I plays crucial roles on neoantigen presentation in tumor cells and the disruption of HLA-I expression may result in immune escape and tumor progression. In this study, we investigated tumor HLA-I from the perspective of allele-specific expression (ASE). To evaluate this phenomenon, we developed a pipeline to detect HLA-I ASE loss based on RNA-seq and WES data. As the expression imbalance was measured on the RNA level, our study is complementary to existing research that focuses on the loss of HLA-I germline heterozygosity (LOH) on the DNA level [12, 29, 42]. The viewpoint of allele-specific expression also differs from the studies in which the total HLA-I expression was considered [43].

Our work demonstrates that HLA-I ASE loss is a frequent phenomenon across tumor types. Moreover, a large proportion of ASE loss cases may not necessarily result from somatic DNA deletions in HLA-I. In terms of impact on overall survival, HLA-I ASE loss did not show a significant association in the pan-cancer analysis, while we noticed that age at diagnosis, tumor ploidy, and macrophage infiltration showed significant trends towards shorter overall survival. This is consistent with the general belief that these factors may play a role in the dynamics of T-cell-directed anti-tumor responses. Despite the non-significant result in pan-cancer analysis, HLA-I ASE loss did have a significant association with prognosis in the basal-like PDAC subtype, as well as in metastatic melanoma under immune checkpoint blockade therapy.

As far as we know, this is the first comprehensive study quantifying the pervasiveness of HLA-I ASE in cancer. Further studies are anticipated to fully elucidate the transcriptional and epigenetic mechanisms driving this phenomenon. We also outline several paths for further investigation into the loss of HLA-I ASE. For instance, studies can be performed to evaluate how the overall survival is impacted by the number of neoantigens with binding affinity to the kept or lost HLA-I allele for each tumor type. Second, with more detailed clinical data, the impact on prognosis should be re-assessed in terms of progression-free survival, an alternative approach to capture the effect of treatment. Moreover, the relationship between HLA-I ASE loss and missense mutations in HLA, including the domains where missense mutations occur (peptide binding groove, TCR interaction site, trans-membrane domain, etc.), can be explored further.

In sum, the prevalence of ASE loss and the initial clinical impact that we have established here highlight the importance for further investigations on the role of HLA-I ASE in cancer, and the necessity of understanding the underlying mechanisms and the timing of this potentially reversible lesion in tumor evolution.

## Conclusions

In conclusion, we have studied the loss of heterogeneity of HLA-I genes across cancer types at the transcriptomic level. Using *arcasHLA-quant*, we inferred the allele-specific expression of HLA-I genes in the tumor and found that HLA-I ASE loss is pervasive across cancer types, with or without detectable somatic DNA lesions. Moreover, we showed that HLA-I ASE loss is significantly associated with worse overall survival in pancreatic ductal adenocarcinoma with basal-like transcriptional subtype using TCGA data and an independent cohort from CUMC. Finally, a retrospective analysis on anti-PD-1 immunotherapy-treated metastatic melanomas revealed an association between HLA-I ASE loss and poor prognosis. Our findings highlight the necessity of further investigation on the roles that HLA-I ASE loss plays in human cancer and immunotherapy treatment.

## Supplementary Information

**Additional file 1.** Additional figures from the results of HLA-I ASE loss calling and survival analysis. **Figure S1.** Agreement between *arcasHLA-quant* and RSEM on HLA-I gene expression quantification for TCGA cohorts. **Figure S2.** Agreement between *arcasHLA-quant* and HLApers on HLA-gene expression quantification for TCGA-PDAC. **Figure S3.** Comparison between *arcasHLA-quant* and HLApers on minor allele frequency calculation in TCGA-PDAC cohort. **Figure S4.** Receiver operating curve of HLA-I ASE loss detection. **Figure S5.** Numbers of cases involved in the analysis in each TCGA cohort. **Figure S6.** Balance of ASE loss across the three HLA-I genes. **Figure S7.** Error bar plots of the log hazard ratios and 95% confidence intervals for the features used in the stratified Cox-regression analysis of overall survival in TCGA cohorts. **Figure S8.** Association between HLA-I ASE loss and overall survival per TCGA cohort. **Figure S9.** Survival curves in TCGA-PDAC data cohort, where cases were divided into two groups based on HLA-I ASE loss status and simultaneous detection of neoantigens with predicted affinity towards genes subject to ASE loss. **Figure S10.** Survival curves in TCGA-PDAC data cohort, where cases are divided into two groups based on HLA-I ASE status. **Figure S11.** HLA-I ASE loss impact on overall survival in TCGA-PDAC by transcriptome-based subtype according to Collisson *et al.*, 2011. **Figure S12.** HLA-I ASE loss impact on overall survival in TCGA-PDAC by transcriptome-based subtype according to Bailey *et al*., 2016. **Figure S13.** Survival curves of TCGA-PDAC according to tumor stages. **Figure S14.** Impact of HLA-I ASE loss on overall survival in CUMC-Epithelial compartment and CUMC-Stroma compartment cohorts. **Figure S15.** Survival curves of TCGA-KIRP cases with purity > 0.1 and being further stratified according to subtypes (identified as P-e.1a, P-e.1b, P-e.2 and P.CIMP-e) or stages (I, II, III and IV). **Figure S16.** Association between HLA-I homozygosity and overall survival in Riaz *et al.*, 2017. **Figure S17.** Refined analysis with neoantigen affinity towards lost HLA alleles increases survival curve separation for fully heterogeneous individuals on-therapy in Riaz *et al.*, 2017. **Figure S18.** HLA-I ASE loss decreases efficacy of immunotherapy in metastatic melanoma. **Figure S19.** Survival associations with sample purity across the patient groups as defined in Fig. 4.

**Additional file 2.** Additional tables from the results of the association between HLA-I ASE loss and microsatellite instability, nonsense and missense mutations. **Table S1.** TCGA case numbers broken down by microsatellite instability and HLA-I ASE loss status. **Table S2.** Association between HLA-I ASE loss and nonsense mutations (a), HLA-I missense mutations (b), contact residue nonsense mutations in HLA-I genes (c) and nonsense/missense B2M mutations (d).

## Data Availability

TCGA data was accessed through dbGaP accession phs000178.v11.p8. Information about TCGA can be found at http://cancergenome.nih.gov. The results published here are partially based upon data generated by The Cancer Genome Atlas (TCGA) managed by the NCI and NHGRI. The CUMC cohort samples were procured by the HICCC Molecular Pathology Share Resource. Outcome data for the CUMC cohort were curated by the HICCC Database Shared Resource. The tool *arcasHLA-quant* and key results of this study are publicly available at https://github.com/RabadanLab/arcasHLA [26].
