# Supplementary material for: Pervasiveness of HLA allele-specific expression loss across tumor types
Source: Genome Med. 2023 Feb 9;15:8. doi: 10.1186/s13073-023-01154-x (PMC9912643; doi:10.1186/s13073-023-01154-x)
Supplement: Supplementary file 1 — Additional file 1. Additional figures from the results of HLA-I ASE loss calling and survival analysis. Figure S1. Agreement between arcasHLA-quant and RSEM on HLA-I gene expression quantification for TCGA cohorts. Figure S2. Agreement between arcasHLA-quant and HLApers on HLA-gene expression quantification for TCGA-PDAC. Figure S3. Comparison between arcasHLA-quant and HLApers on minor allele frequency calculation in TCGA-PDAC cohort. Figure S4. Receiver operating curve of HLA-I ASE loss detection. Figure S5. Numbers of cases involved in the analysis in each TCGA cohort. Figure S6. Balance of ASE loss across the three HLA-I genes. Figure S7. Error bar plots of the log hazard ratios and 95% confidence intervals for the features used in the stratified Cox-regression analysis of overall survival in TCGA cohorts. Figure S8. Association between HLA-I ASE loss and overall survival per TCGA cohort. Figure S9. Survival curves in TCGA-PDAC data cohort, where cases were divided into two groups based on HLA-I ASE loss status and simultaneous detection of neoantigens with predicted affinity towards genes subject to ASE loss. Figure S10. Survival curves in TCGA-PDAC data cohort, where cases are divided into two groups based on HLA-I ASE status. Figure S11. HLA-I ASE loss impact on overall survival in TCGA-PDAC by transcriptome-based subtype according to Collisson et al., 2011. Figure S12. HLA-I ASE loss impact on overall survival in TCGA-PDAC by transcriptome-based subtype according to Bailey et al., 2016. Figure S13. Survival curves of TCGA-PDAC according to tumor stages. Figure S14. Impact of HLA-I ASE loss on overall survival in CUMC-Epithelial compartment and CUMC-Stroma compartment cohorts. Figure S15. Survival curves of TCGA-KIRP cases with purity > 0.1 and being further stratified according to subtypes (identified as P-e.1a, P-e.1b, P-e.2 and P.CIMP-e) or stages (I, II, III and IV). Figure S16. Association between HLA-I homozygosity and overall survival in Riaz et al., 2 [file 13073_2023_1154_MOESM1_ESM.pptx]

## Slide 1
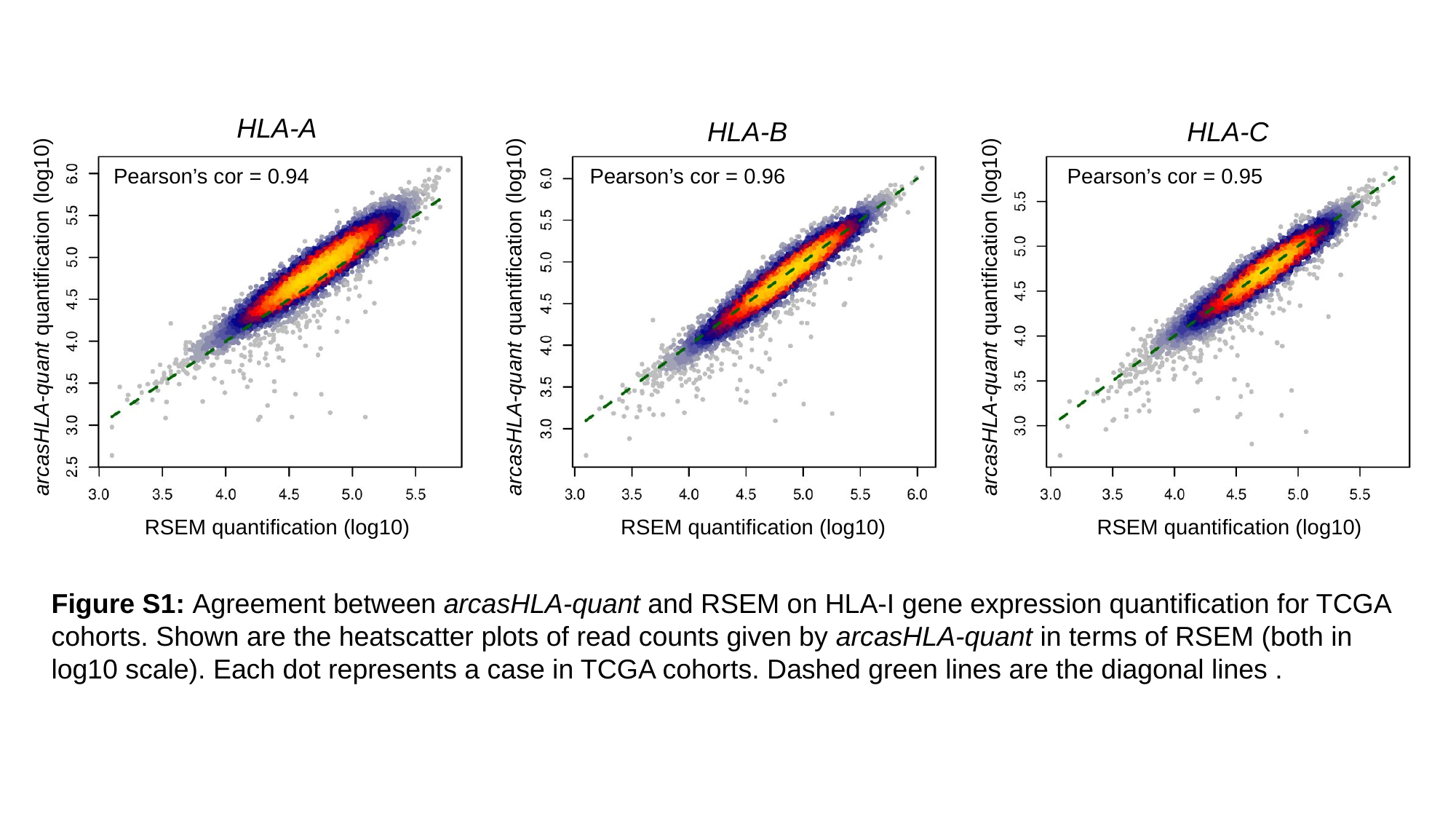

HLA-A
HLA-B
HLA-C
Pearson’s cor = 0.94
Pearson’s cor = 0.96
Pearson’s cor = 0.95
arcasHLA-quant quantification (log10)
arcasHLA-quant quantification (log10)
arcasHLA-quant quantification (log10)
RSEM quantification (log10)
RSEM quantification (log10)
RSEM quantification (log10)

## Slide 2
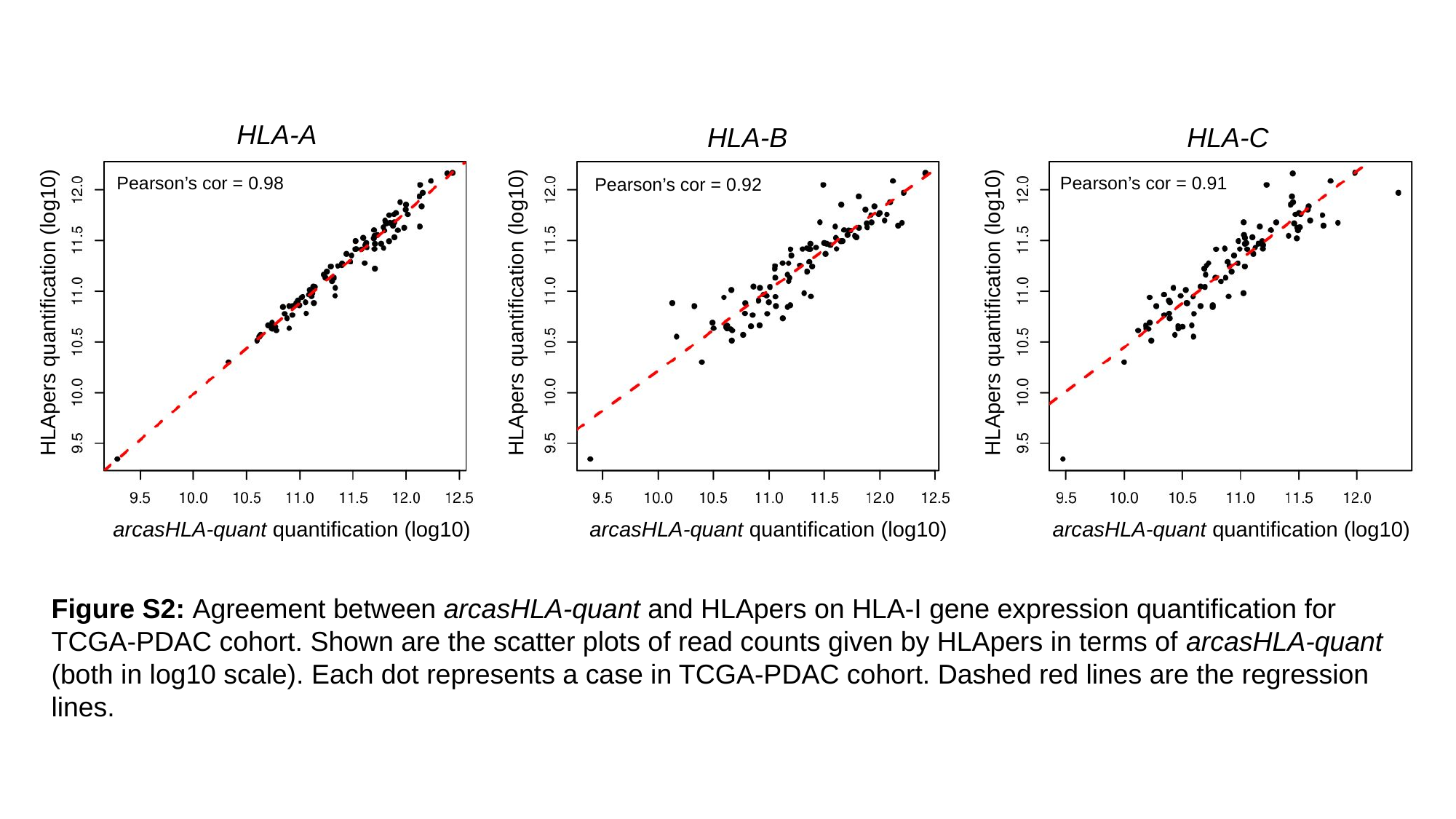

HLA-A
HLA-B
HLA-C
Pearson’s cor = 0.98
Pearson’s cor = 0.91
Pearson’s cor = 0.92
HLApers quantification (log10)
HLApers quantification (log10)
HLApers quantification (log10)
arcasHLA-quant quantification (log10)
arcasHLA-quant quantification (log10)
arcasHLA-quant quantification (log10)
Figure S2: Agreement between arcasHLA-quant and HLApers on HLA-I gene expression quantification for TCGA-PDAC cohort. Shown are the scatter plots of read counts given by HLApers in terms of arcasHLA-quant (both in log10 scale). Each dot represents a case in TCGA-PDAC cohort. Dashed red lines are the regression lines.

## Slide 3
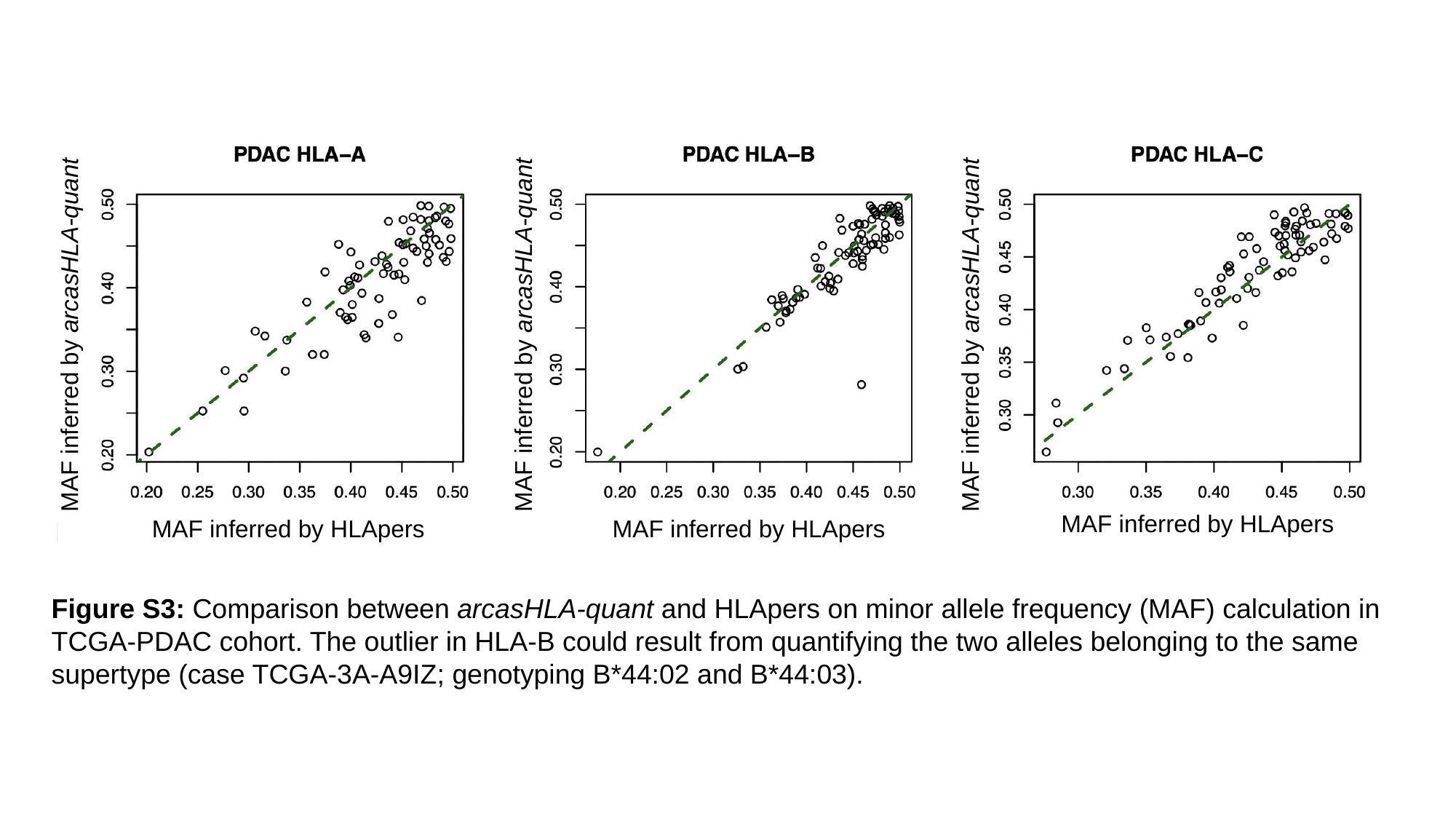

MAF inferred by arcasHLA-quant
MAF inferred by arcasHLA-quant
MAF inferred by arcasHLA-quant
MAF inferred by HLApers
MAF inferred by HLApers
MAF inferred by HLApers
Figure S3: Comparison between arcasHLA-quant and HLApers on minor allele frequency (MAF) calculation in TCGA-PDAC cohort. The outlier in HLA-B could result from quantifying the two alleles belonging to the same supertype (case TCGA-3A-A9IZ; genotyping B*44:02 and B*44:03).

## Slide 4
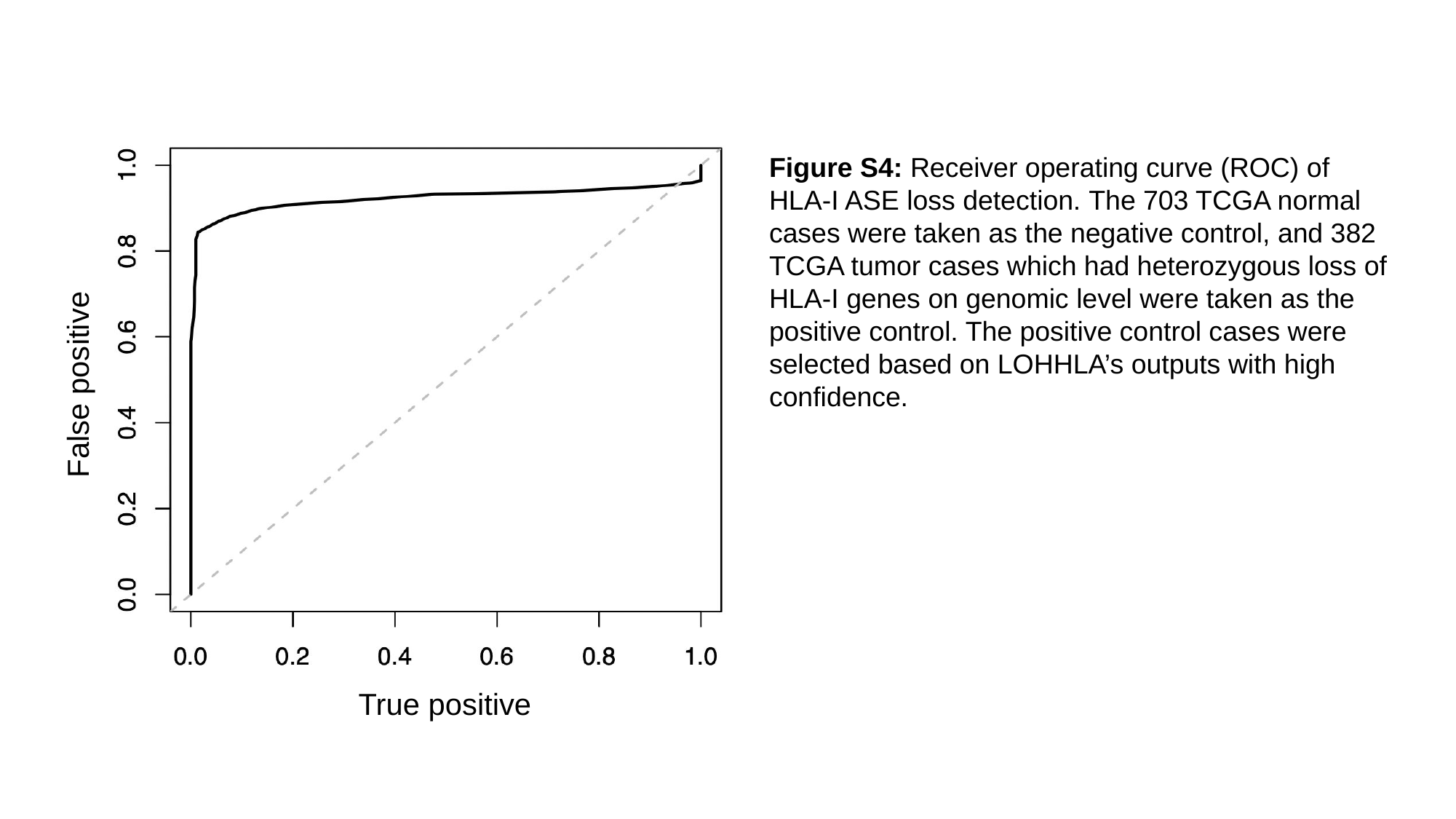

Figure S4: Receiver operating curve (ROC) of HLA-I ASE loss detection. The 703 TCGA normal cases were taken as the negative control, and 382 TCGA tumor cases which had heterozygous loss of HLA-I genes on genomic level were taken as the positive control. The positive control cases were selected based on LOHHLA’s outputs with high confidence.
False positive
True positive

## Slide 5
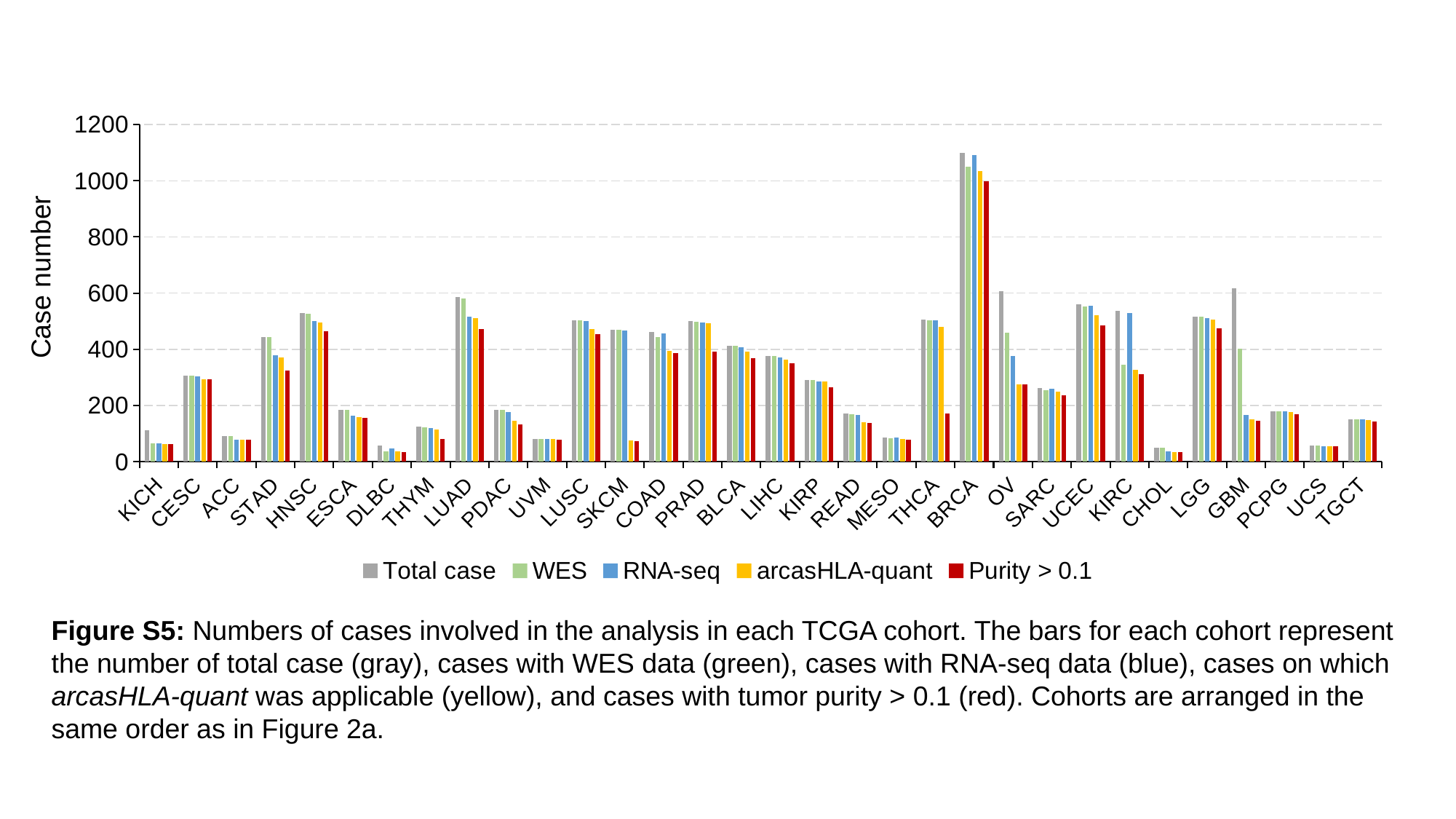

### Chart
| Category | Total case | WES | RNA-seq | arcasHLA-quant | Purity > 0.1 |
|---|---|---|---|---|---|
| KICH | 113.0 | 66.0 | 66.0 | 64.0 | 63.0 |
| CESC | 307.0 | 305.0 | 304.0 | 294.0 | 294.0 |
| ACC | 92.0 | 92.0 | 79.0 | 78.0 | 78.0 |
| STAD | 443.0 | 443.0 | 380.0 | 371.0 | 324.0 |
| HNSC | 528.0 | 527.0 | 501.0 | 495.0 | 465.0 |
| ESCA | 185.0 | 184.0 | 164.0 | 158.0 | 155.0 |
| DLBC | 58.0 | 37.0 | 48.0 | 37.0 | 35.0 |
| THYM | 124.0 | 123.0 | 119.0 | 115.0 | 81.0 |
| LUAD | 585.0 | 582.0 | 515.0 | 511.0 | 472.0 |
| PDAC | 185.0 | 185.0 | 177.0 | 146.0 | 133.0 |
| UVM | 80.0 | 80.0 | 80.0 | 80.0 | 79.0 |
| LUSC | 504.0 | 502.0 | 501.0 | 472.0 | 453.0 |
| SKCM | 470.0 | 470.0 | 468.0 | 76.0 | 73.0 |
| COAD | 461.0 | 443.0 | 456.0 | 394.0 | 386.0 |
| PRAD | 500.0 | 498.0 | 496.0 | 492.0 | 392.0 |
| BLCA | 412.0 | 412.0 | 408.0 | 391.0 | 369.0 |
| LIHC | 377.0 | 376.0 | 371.0 | 363.0 | 351.0 |
| KIRP | 291.0 | 290.0 | 285.0 | 285.0 | 266.0 |
| READ | 172.0 | 168.0 | 167.0 | 140.0 | 139.0 |
| MESO | 87.0 | 83.0 | 86.0 | 82.0 | 78.0 |
| THCA | 507.0 | 502.0 | 502.0 | 481.0 | 172.0 |
| BRCA | 1098.0 | 1050.0 | 1092.0 | 1034.0 | 999.0 |
| OV | 608.0 | 460.0 | 376.0 | 274.0 | 274.0 |
| SARC | 261.0 | 255.0 | 259.0 | 248.0 | 236.0 |
| UCEC | 560.0 | 553.0 | 555.0 | 521.0 | 484.0 |
| KIRC | 537.0 | 345.0 | 530.0 | 326.0 | 311.0 |
| CHOL | 51.0 | 51.0 | 36.0 | 34.0 | 33.0 |
| LGG | 516.0 | 515.0 | 512.0 | 505.0 | 474.0 |
| GBM | 617.0 | 401.0 | 166.0 | 151.0 | 146.0 |
| PCPG | 179.0 | 179.0 | 179.0 | 177.0 | 168.0 |
| UCS | 57.0 | 57.0 | 56.0 | 56.0 | 55.0 |
| TGCT | 150.0 | 150.0 | 150.0 | 149.0 | 144.0 |Case number
Figure S5: Numbers of cases involved in the analysis in each TCGA cohort. The bars for each cohort represent the number of total case (gray), cases with WES data (green), cases with RNA-seq data (blue), cases on which arcasHLA-quant was applicable (yellow), and cases with tumor purity > 0.1 (red). Cohorts are arranged in the same order as in Figure 2a.

## Slide 6
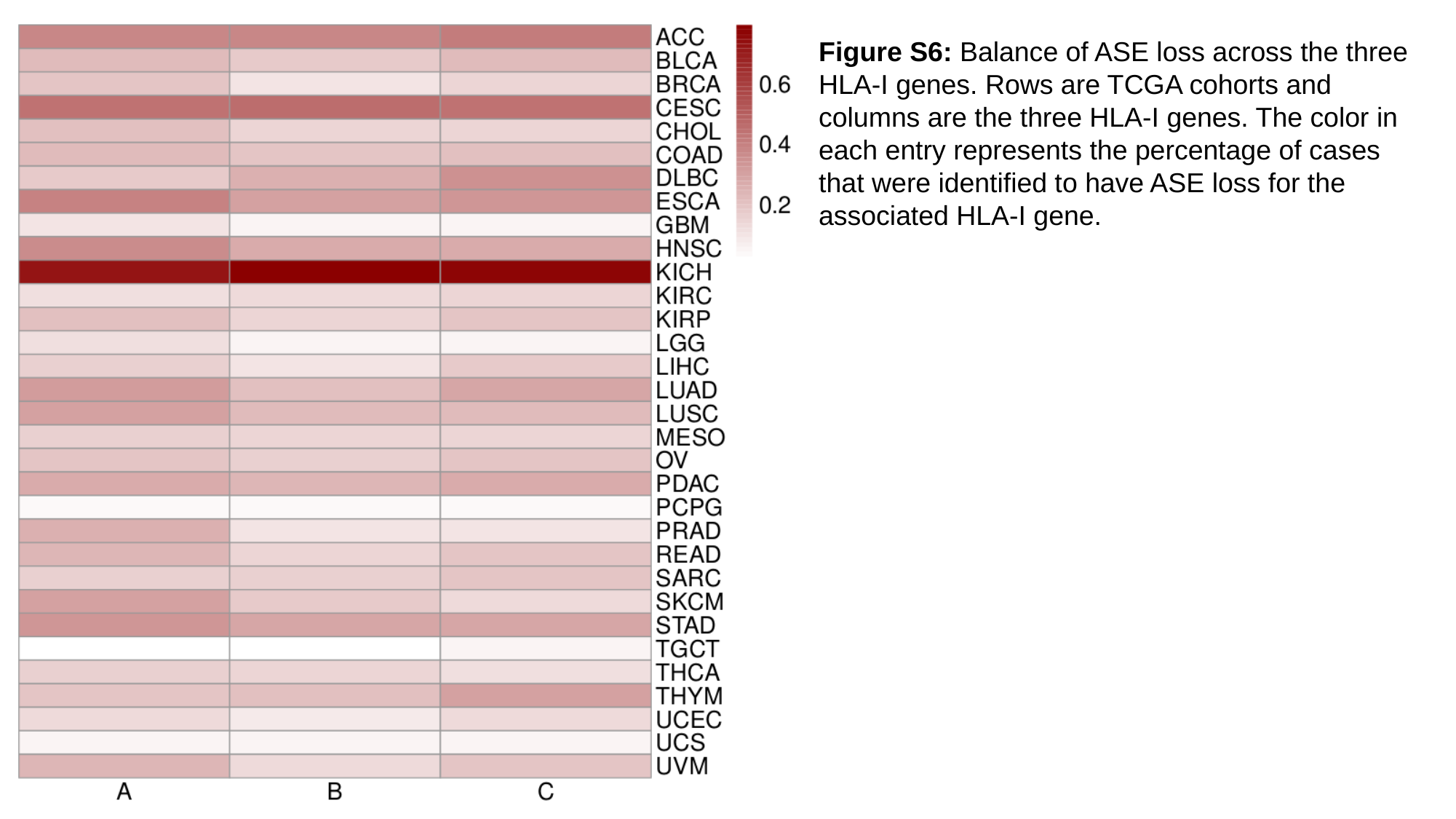

Figure S6: Balance of ASE loss across the three HLA-I genes. Rows are TCGA cohorts and columns are the three HLA-I genes. The color in each entry represents the percentage of cases that were identified to have ASE loss for the associated HLA-I gene.

## Slide 7
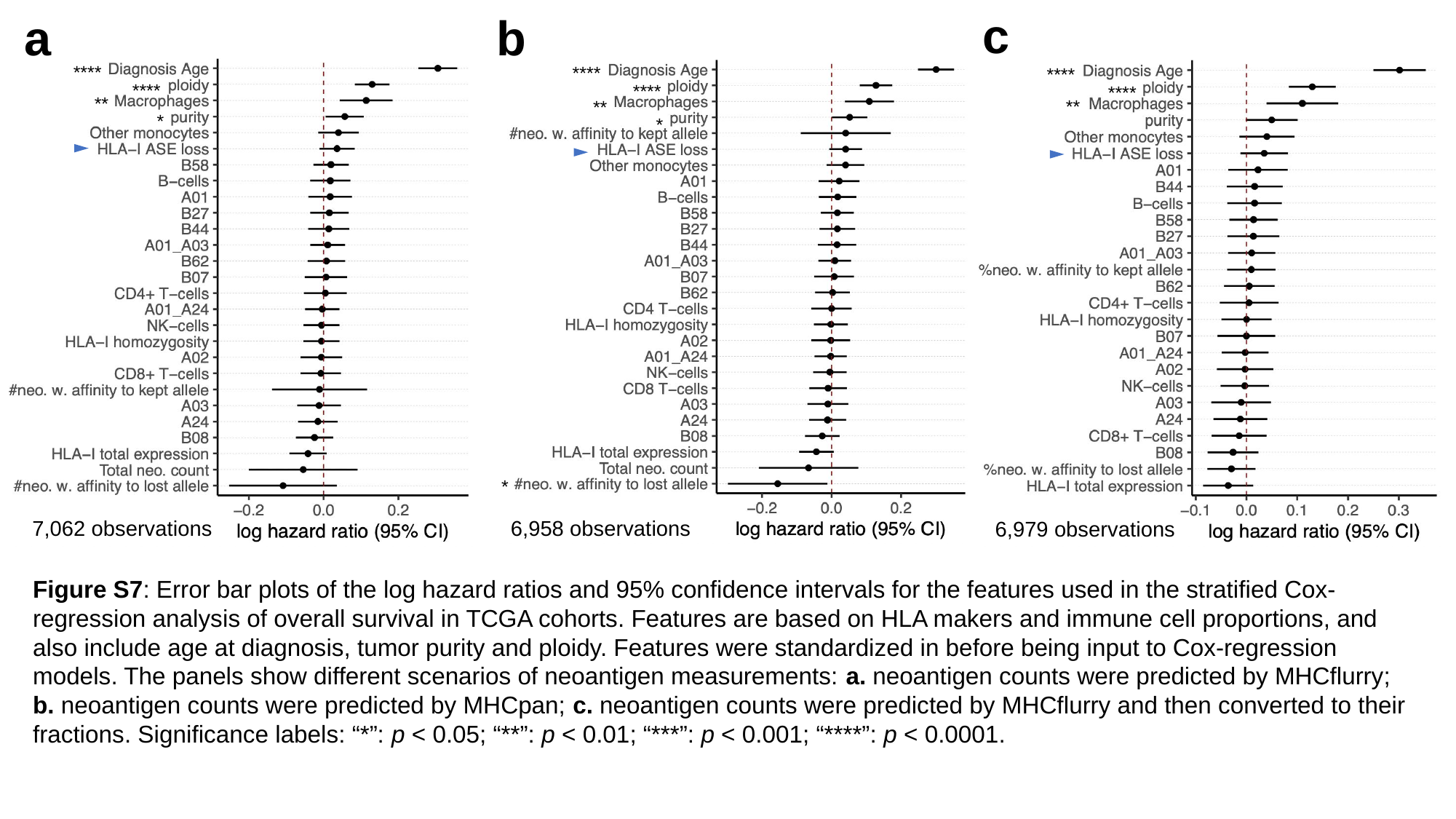

c
a
b
****
****
****
****
****
****
**
**
**
*
*
*
7,062 observations
6,958 observations
6,979 observations
Figure S7: Error bar plots of the log hazard ratios and 95% confidence intervals for the features used in the stratified Cox-regression analysis of overall survival in TCGA cohorts. Features are based on HLA makers and immune cell proportions, and also include age at diagnosis, tumor purity and ploidy. Features were standardized in before being input to Cox-regression models. The panels show different scenarios of neoantigen measurements: a. neoantigen counts were predicted by MHCflurry; b. neoantigen counts were predicted by MHCpan; c. neoantigen counts were predicted by MHCflurry and then converted to their fractions. Significance labels: “*”: p < 0.05; “**”: p < 0.01; “***”: p < 0.001; “****”: p < 0.0001.

## Slide 8
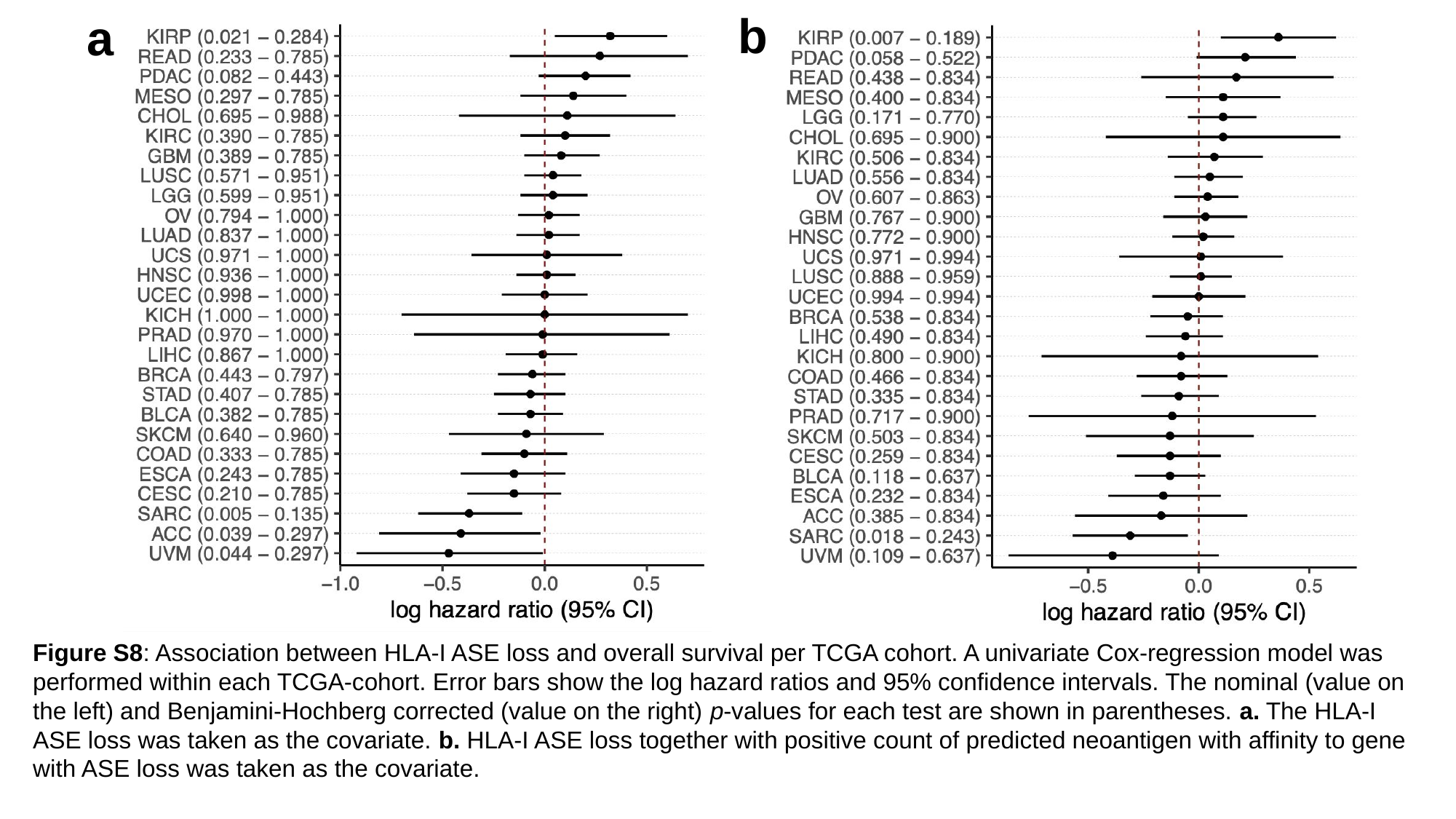

b
a
Figure S8: Association between HLA-I ASE loss and overall survival per TCGA cohort. A univariate Cox-regression model was performed within each TCGA-cohort. Error bars show the log hazard ratios and 95% confidence intervals. The nominal (value on the left) and Benjamini-Hochberg corrected (value on the right) p-values for each test are shown in parentheses. a. The HLA-I ASE loss was taken as the covariate. b. HLA-I ASE loss together with positive count of predicted neoantigen with affinity to gene with ASE loss was taken as the covariate.

## Slide 9
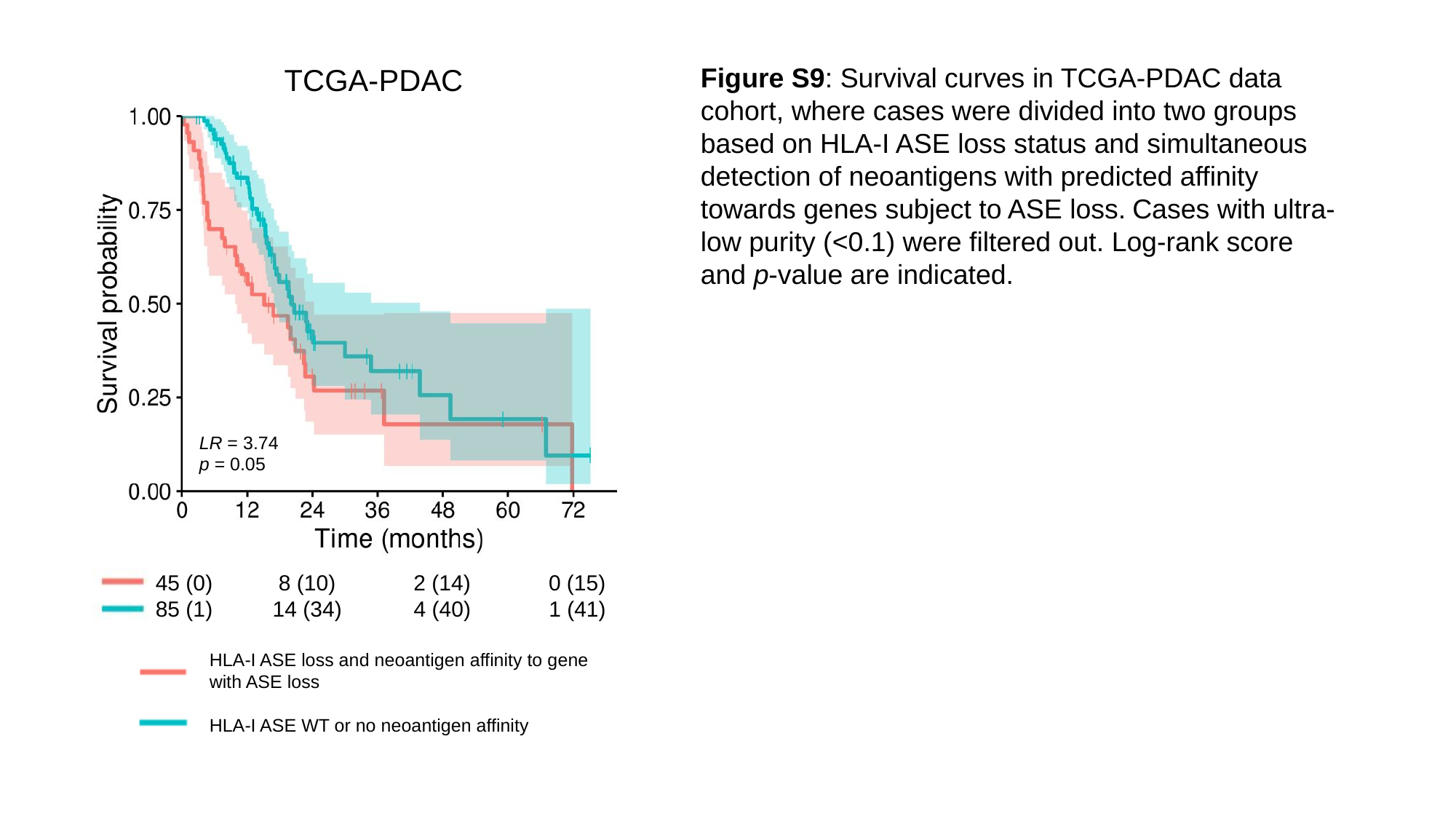

TCGA-PDAC
Figure S9: Survival curves in TCGA-PDAC data cohort, where cases were divided into two groups based on HLA-I ASE loss status and simultaneous detection of neoantigens with predicted affinity towards genes subject to ASE loss. Cases with ultra-low purity (<0.1) were filtered out. Log-rank score and p-value are indicated.
LR = 3.74
p = 0.05
45 (0) 8 (10) 2 (14) 0 (15)
85 (1) 14 (34) 4 (40) 1 (41)
HLA-I ASE loss and neoantigen affinity to gene with ASE loss
HLA-I ASE WT or no neoantigen affinity

## Slide 10
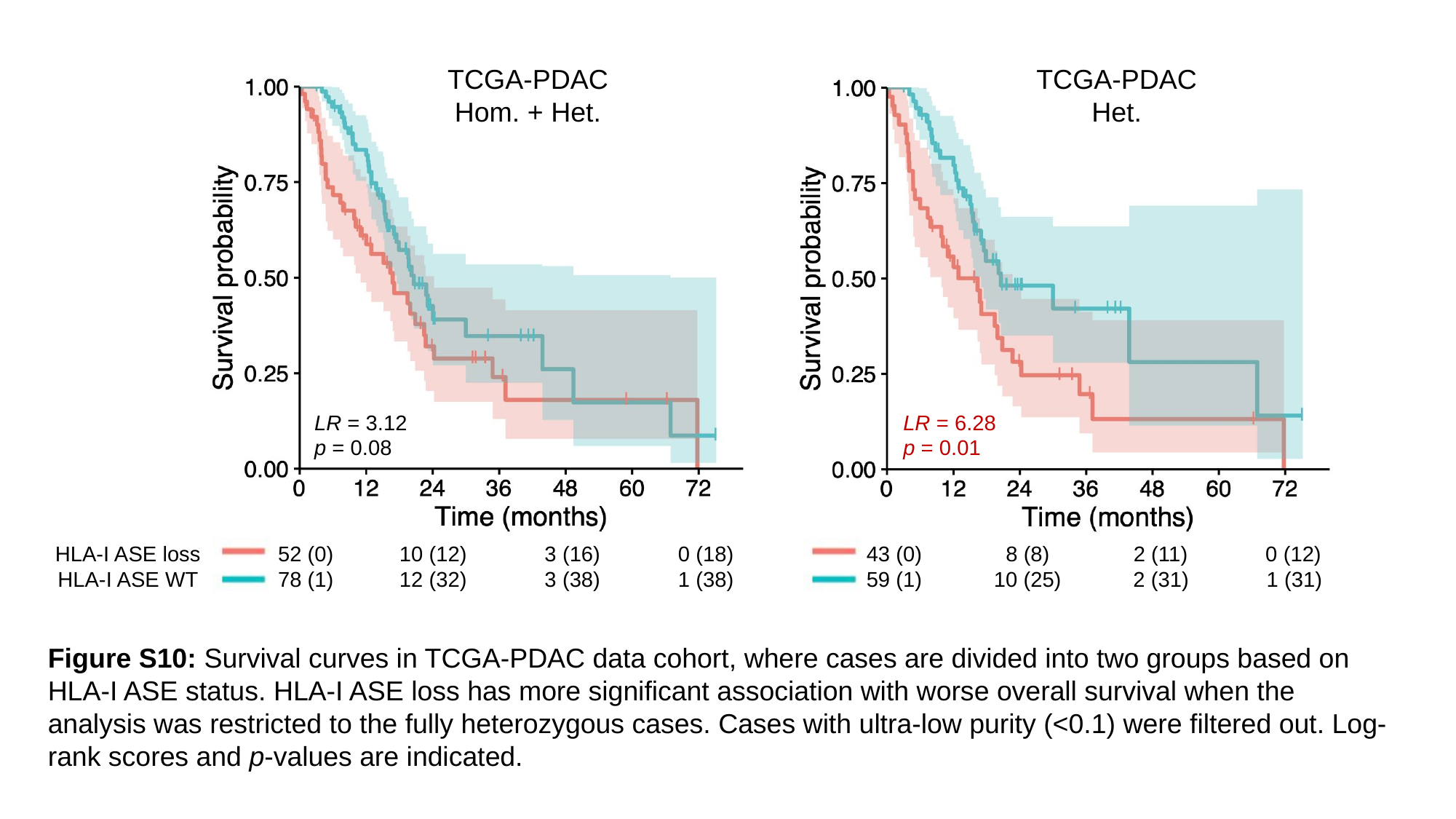

TCGA-PDAC
Hom. + Het.
TCGA-PDAC
Het.
LR = 3.12
p = 0.08
LR = 6.28
p = 0.01
HLA-I ASE loss
HLA-I ASE WT
52 (0) 10 (12) 3 (16) 0 (18)
78 (1) 12 (32) 3 (38) 1 (38)
43 (0) 8 (8) 2 (11) 0 (12)
59 (1) 10 (25) 2 (31) 1 (31)
Figure S10: Survival curves in TCGA-PDAC data cohort, where cases are divided into two groups based on HLA-I ASE status. HLA-I ASE loss has more significant association with worse overall survival when the analysis was restricted to the fully heterozygous cases. Cases with ultra-low purity (<0.1) were filtered out. Log-rank scores and p-values are indicated.

## Slide 11
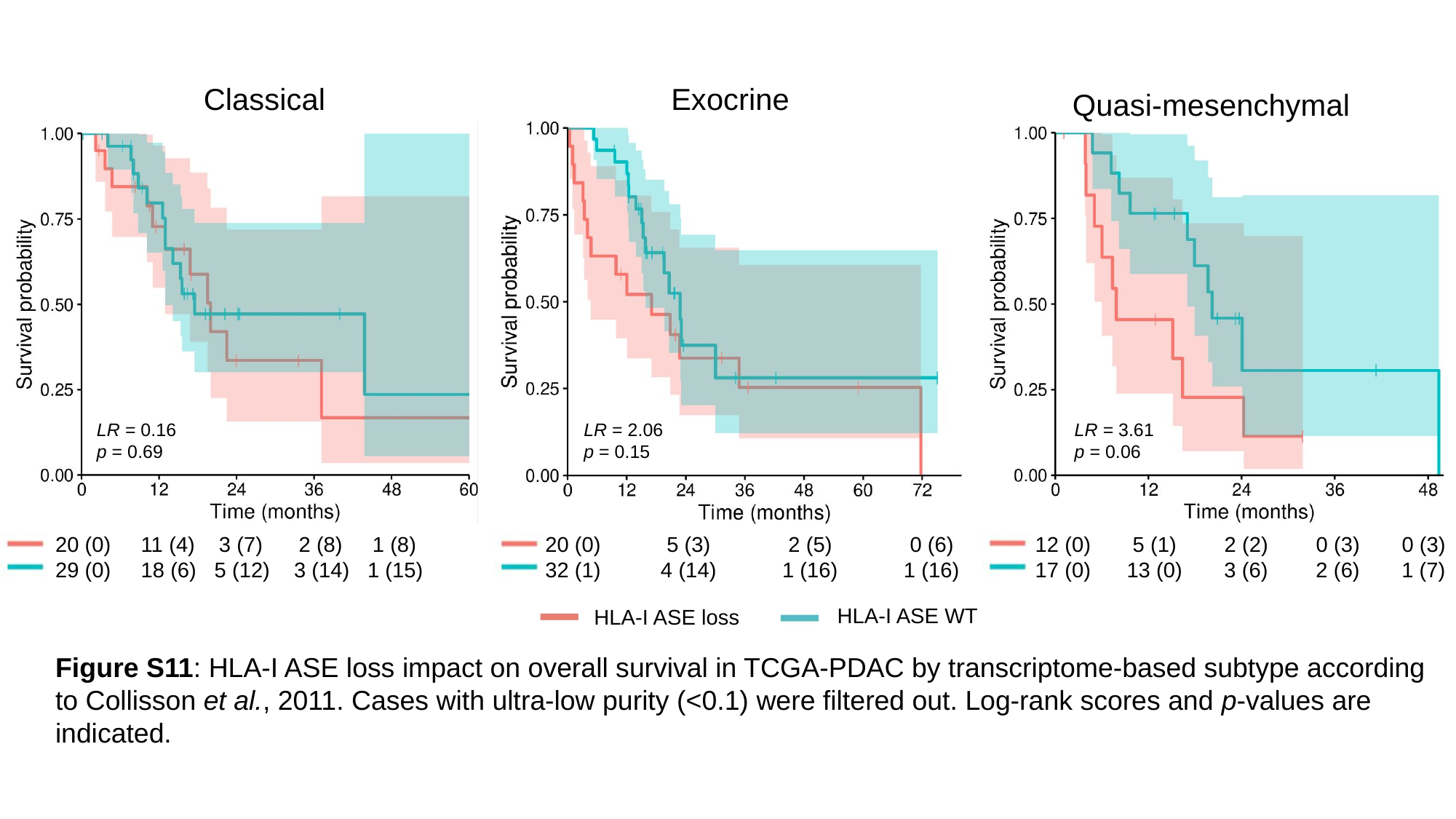

Classical
Exocrine
Quasi-mesenchymal
LR = 2.06
p = 0.15
LR = 3.61
p = 0.06
LR = 0.16
p = 0.69
20 (0) 11 (4) 3 (7) 2 (8) 1 (8)
29 (0) 18 (6) 5 (12) 3 (14) 1 (15)
20 (0) 5 (3) 2 (5) 0 (6)
32 (1) 4 (14) 1 (16) 1 (16)
12 (0) 5 (1) 2 (2) 0 (3) 0 (3)
17 (0) 13 (0) 3 (6) 2 (6) 1 (7)
HLA-I ASE WT
HLA-I ASE loss
Figure S11: HLA-I ASE loss impact on overall survival in TCGA-PDAC by transcriptome-based subtype according to Collisson et al., 2011. Cases with ultra-low purity (<0.1) were filtered out. Log-rank scores and p-values are indicated.

## Slide 12
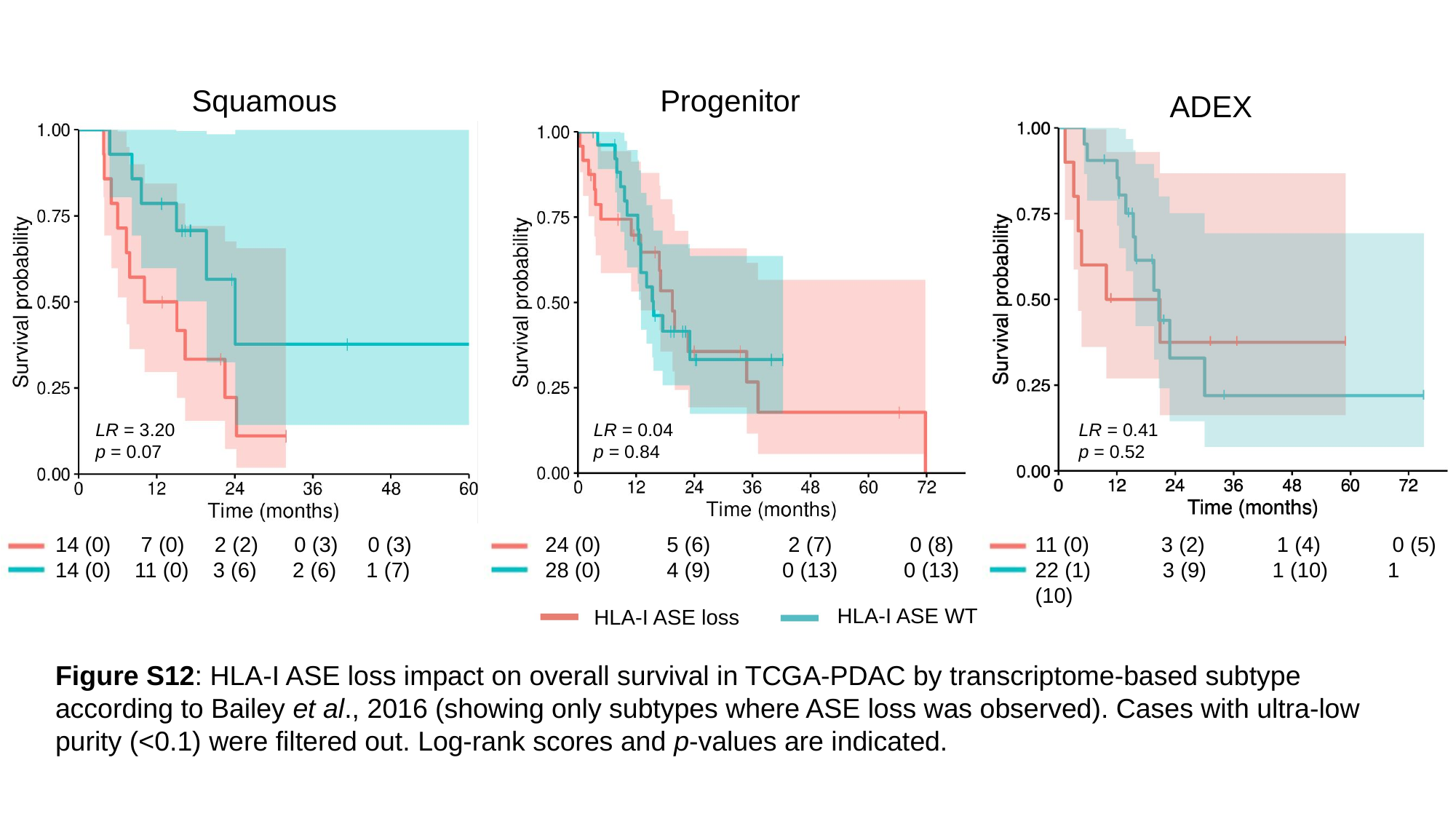

Squamous
Progenitor
ADEX
LR = 0.41
p = 0.52
LR = 0.04
p = 0.84
LR = 3.20
p = 0.07
14 (0) 7 (0) 2 (2) 0 (3) 0 (3)
14 (0) 11 (0) 3 (6) 2 (6) 1 (7)
24 (0) 5 (6) 2 (7) 0 (8)
28 (0) 4 (9) 0 (13) 0 (13)
11 (0) 3 (2) 1 (4) 0 (5)
22 (1) 3 (9) 1 (10) 1 (10)
HLA-I ASE WT
HLA-I ASE loss
Figure S12: HLA-I ASE loss impact on overall survival in TCGA-PDAC by transcriptome-based subtype according to Bailey et al., 2016 (showing only subtypes where ASE loss was observed). Cases with ultra-low purity (<0.1) were filtered out. Log-rank scores and p-values are indicated.

## Slide 13
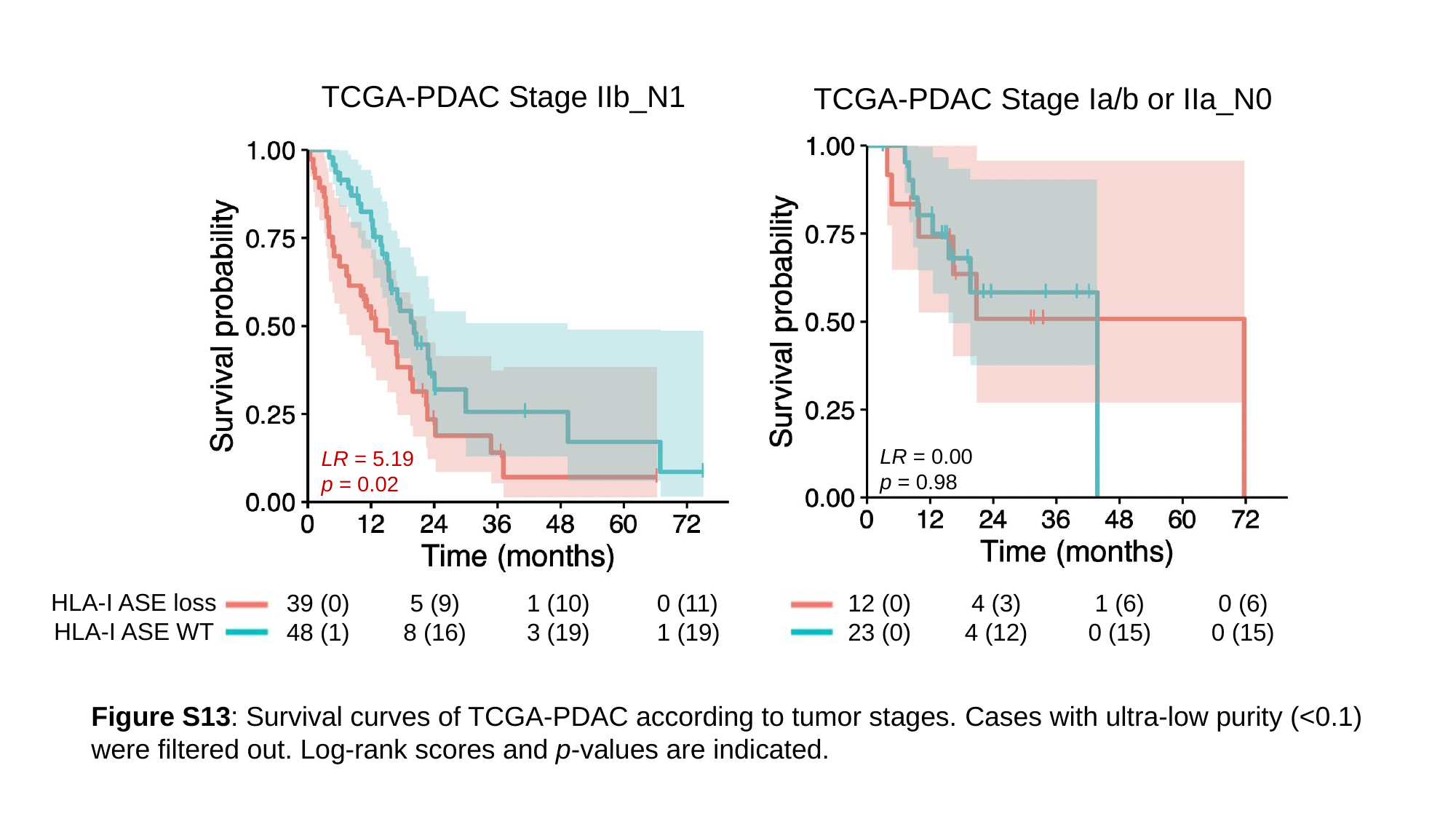

TCGA-PDAC Stage IIb_N1
TCGA-PDAC Stage Ia/b or IIa_N0
LR = 0.00
p = 0.98
LR = 5.19
p = 0.02
HLA-I ASE loss
HLA-I ASE WT
39 (0) 5 (9) 1 (10) 0 (11)
48 (1) 8 (16) 3 (19) 1 (19)
12 (0) 4 (3) 1 (6) 0 (6)
23 (0) 4 (12) 0 (15) 0 (15)
Figure S13: Survival curves of TCGA-PDAC according to tumor stages. Cases with ultra-low purity (<0.1) were filtered out. Log-rank scores and p-values are indicated.

## Slide 14
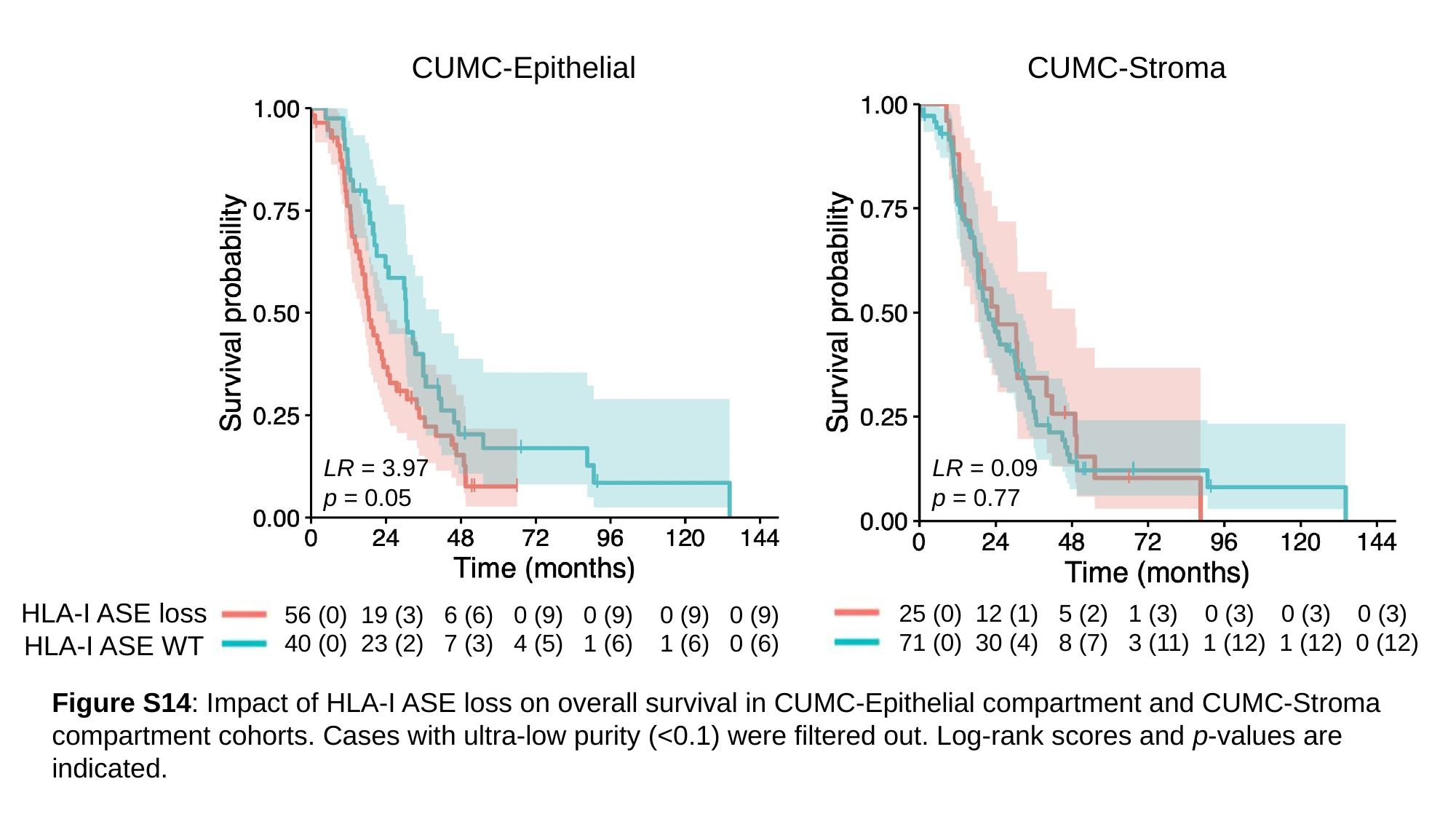

CUMC-Epithelial
CUMC-Stroma
LR = 0.09
p = 0.77
LR = 3.97
p = 0.05
HLA-I ASE loss
HLA-I ASE WT
25 (0) 12 (1) 5 (2) 1 (3) 0 (3) 0 (3) 0 (3)
71 (0) 30 (4) 8 (7) 3 (11) 1 (12) 1 (12) 0 (12)
56 (0) 19 (3) 6 (6) 0 (9) 0 (9) 0 (9) 0 (9)
40 (0) 23 (2) 7 (3) 4 (5) 1 (6) 1 (6) 0 (6)
Figure S14: Impact of HLA-I ASE loss on overall survival in CUMC-Epithelial compartment and CUMC-Stroma compartment cohorts. Cases with ultra-low purity (<0.1) were filtered out. Log-rank scores and p-values are indicated.

## Slide 15
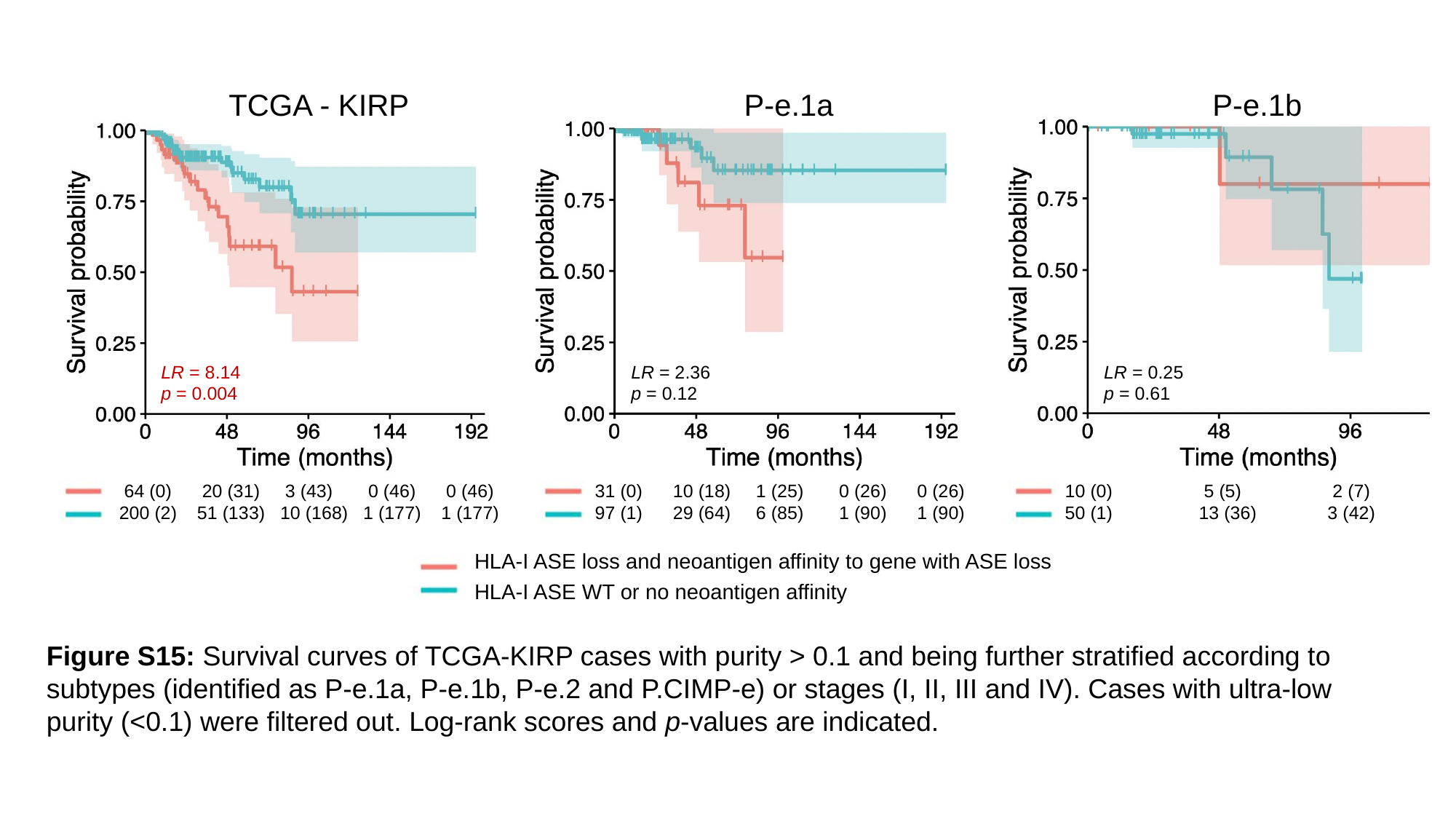

TCGA - KIRP
P-e.1a
P-e.1b
LR = 0.25
p = 0.61
LR = 2.36
p = 0.12
LR = 8.14
p = 0.004
10 (0) 5 (5) 2 (7)
50 (1) 13 (36) 3 (42)
31 (0) 10 (18) 1 (25) 0 (26) 0 (26)
97 (1) 29 (64) 6 (85) 1 (90) 1 (90)
 64 (0) 20 (31) 3 (43) 0 (46) 0 (46)
200 (2) 51 (133) 10 (168) 1 (177) 1 (177)
HLA-I ASE loss and neoantigen affinity to gene with ASE loss
HLA-I ASE WT or no neoantigen affinity
Figure S15: Survival curves of TCGA-KIRP cases with purity > 0.1 and being further stratified according to subtypes (identified as P-e.1a, P-e.1b, P-e.2 and P.CIMP-e) or stages (I, II, III and IV). Cases with ultra-low purity (<0.1) were filtered out. Log-rank scores and p-values are indicated.

## Slide 16
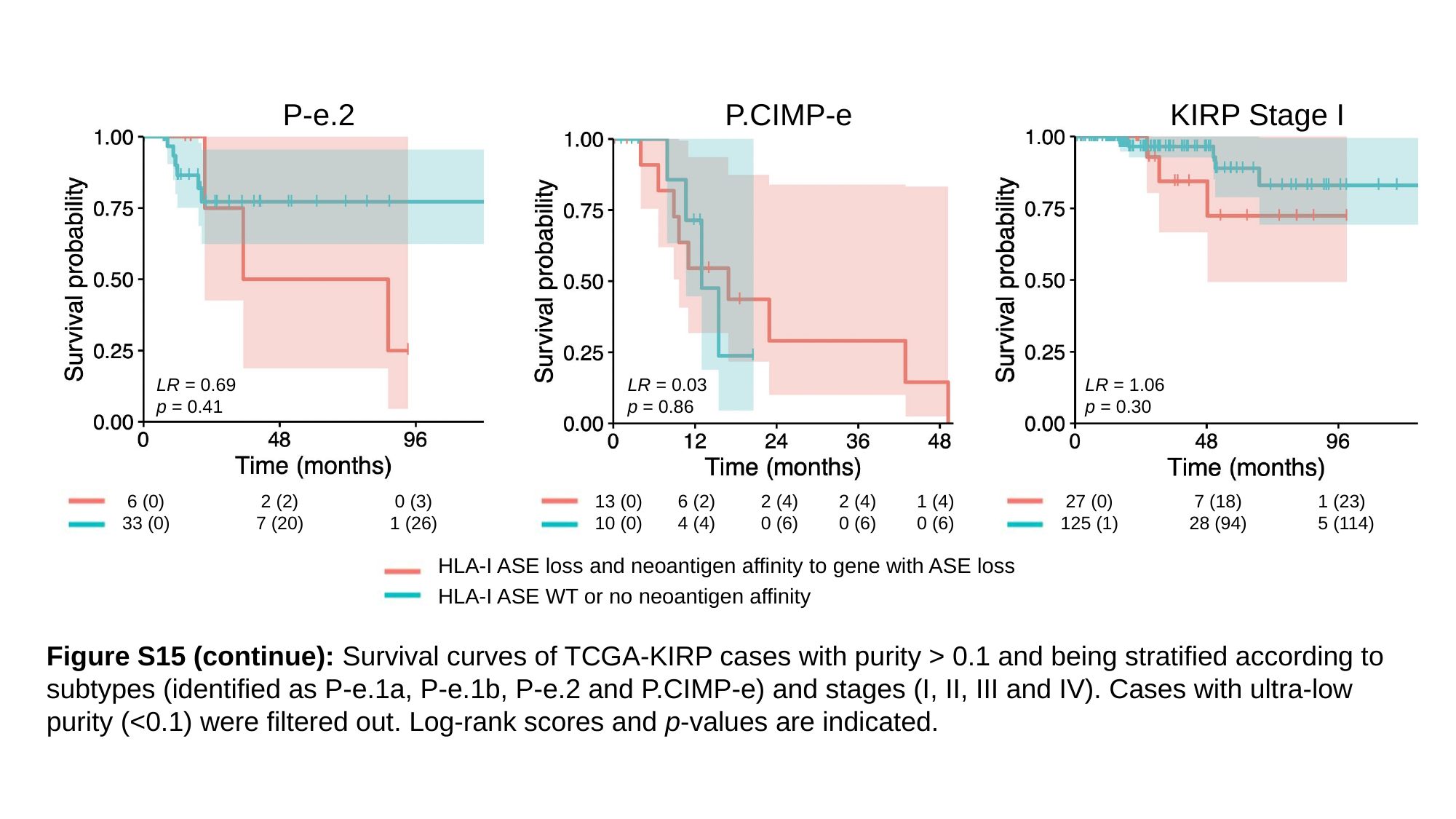

P-e.2
P.CIMP-e
KIRP Stage I
LR = 0.03
p = 0.86
LR = 0.69
p = 0.41
LR = 1.06
p = 0.30
 6 (0) 2 (2) 0 (3)
33 (0) 7 (20) 1 (26)
 27 (0) 7 (18) 1 (23)
125 (1) 28 (94) 5 (114)
13 (0) 6 (2) 2 (4) 2 (4) 1 (4)
10 (0) 4 (4) 0 (6) 0 (6) 0 (6)
HLA-I ASE loss and neoantigen affinity to gene with ASE loss
HLA-I ASE WT or no neoantigen affinity
Figure S15 (continue): Survival curves of TCGA-KIRP cases with purity > 0.1 and being stratified according to subtypes (identified as P-e.1a, P-e.1b, P-e.2 and P.CIMP-e) and stages (I, II, III and IV). Cases with ultra-low purity (<0.1) were filtered out. Log-rank scores and p-values are indicated.

## Slide 17
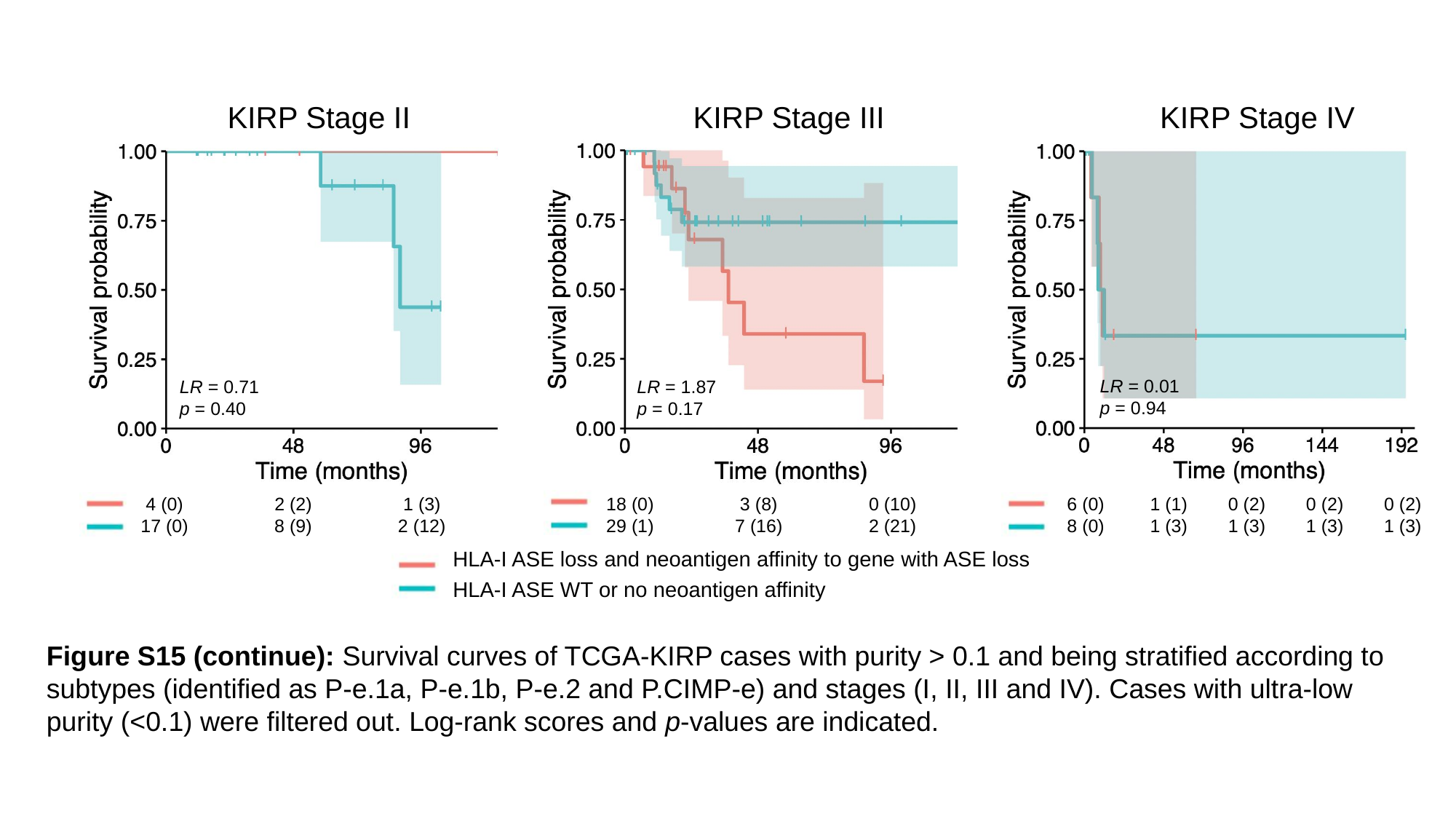

KIRP Stage II
KIRP Stage III
KIRP Stage IV
LR = 0.01
p = 0.94
LR = 0.71
p = 0.40
LR = 1.87
p = 0.17
 4 (0) 2 (2) 1 (3)
17 (0) 8 (9) 2 (12)
18 (0) 3 (8) 0 (10)
29 (1) 7 (16) 2 (21)
6 (0) 1 (1) 0 (2) 0 (2) 0 (2)
8 (0) 1 (3) 1 (3) 1 (3) 1 (3)
HLA-I ASE loss and neoantigen affinity to gene with ASE loss
HLA-I ASE WT or no neoantigen affinity
Figure S15 (continue): Survival curves of TCGA-KIRP cases with purity > 0.1 and being stratified according to subtypes (identified as P-e.1a, P-e.1b, P-e.2 and P.CIMP-e) and stages (I, II, III and IV). Cases with ultra-low purity (<0.1) were filtered out. Log-rank scores and p-values are indicated.

## Slide 18
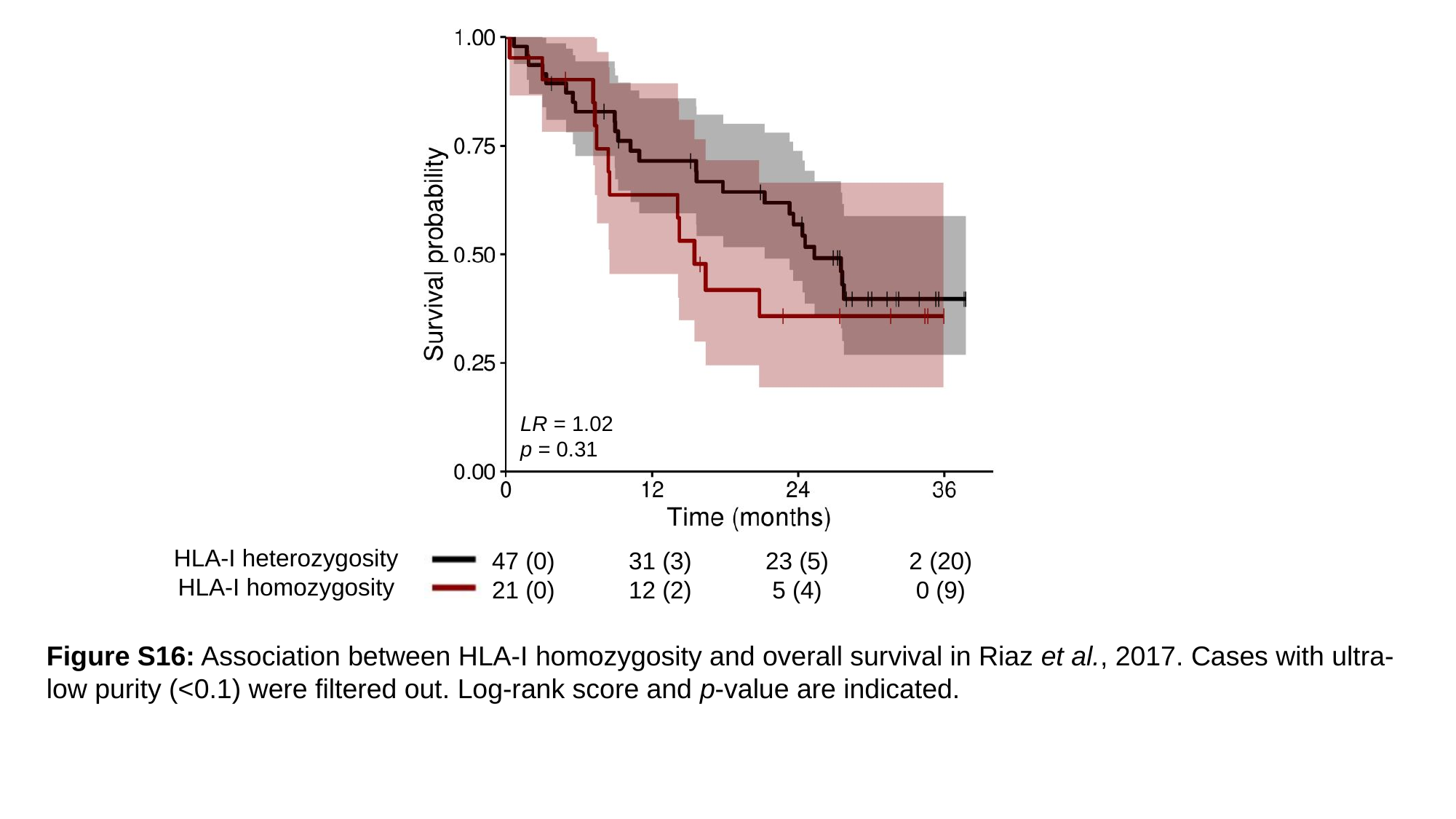

LR = 1.02
p = 0.31
HLA-I heterozygosity
HLA-I homozygosity
47 (0) 31 (3) 23 (5) 2 (20)
21 (0) 12 (2) 5 (4) 0 (9)
Figure S16: Association between HLA-I homozygosity and overall survival in Riaz et al., 2017. Cases with ultra-low purity (<0.1) were filtered out. Log-rank score and p-value are indicated.

## Slide 19
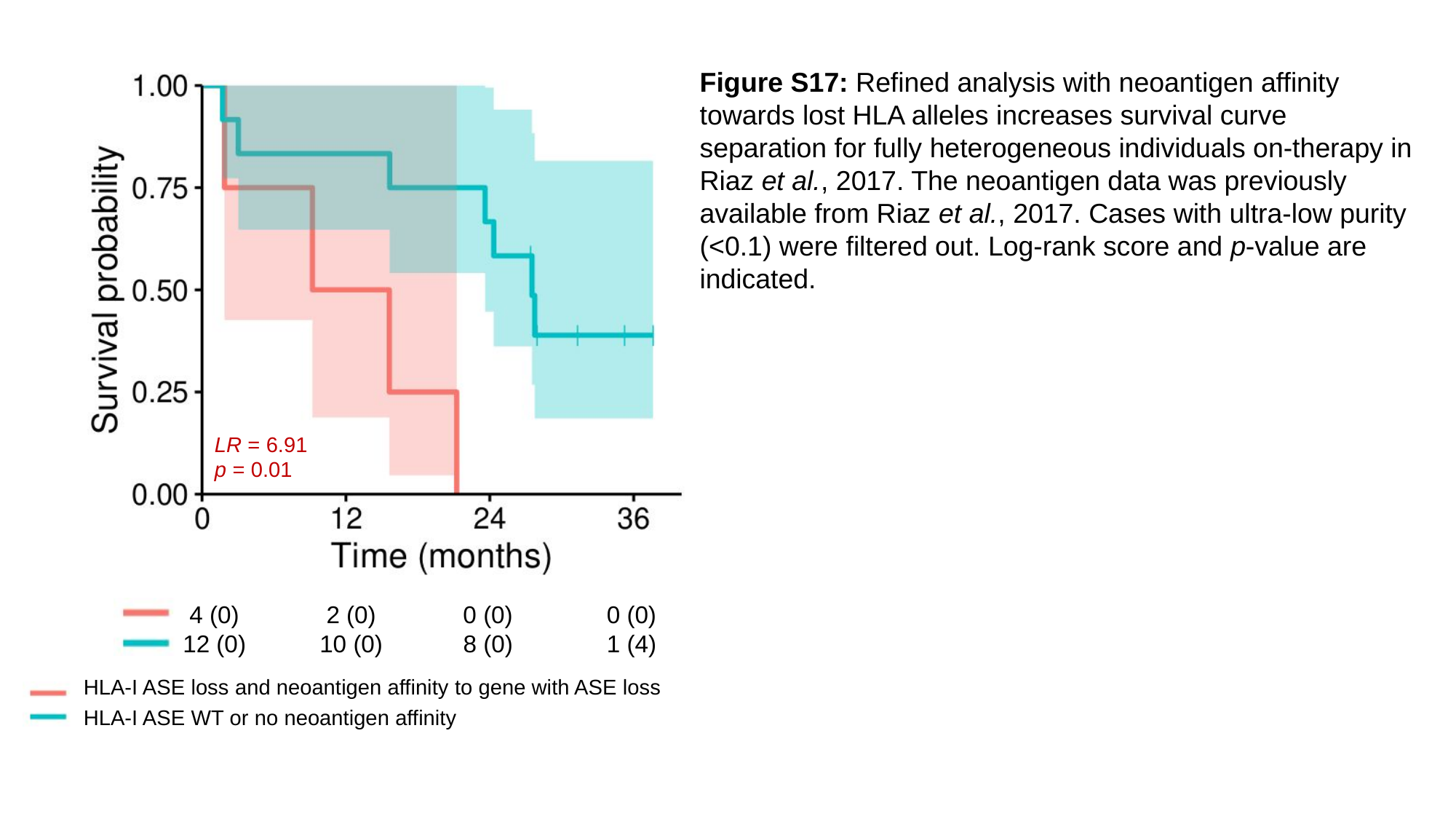

Figure S17: Refined analysis with neoantigen affinity towards lost HLA alleles increases survival curve separation for fully heterogeneous individuals on-therapy in Riaz et al., 2017. The neoantigen data was previously available from Riaz et al., 2017. Cases with ultra-low purity (<0.1) were filtered out. Log-rank score and p-value are indicated.
LR = 6.91
p = 0.01
 4 (0) 2 (0) 0 (0) 0 (0)
12 (0) 10 (0) 8 (0) 1 (4)
HLA-I ASE loss and neoantigen affinity to gene with ASE loss
HLA-I ASE WT or no neoantigen affinity

## Slide 20
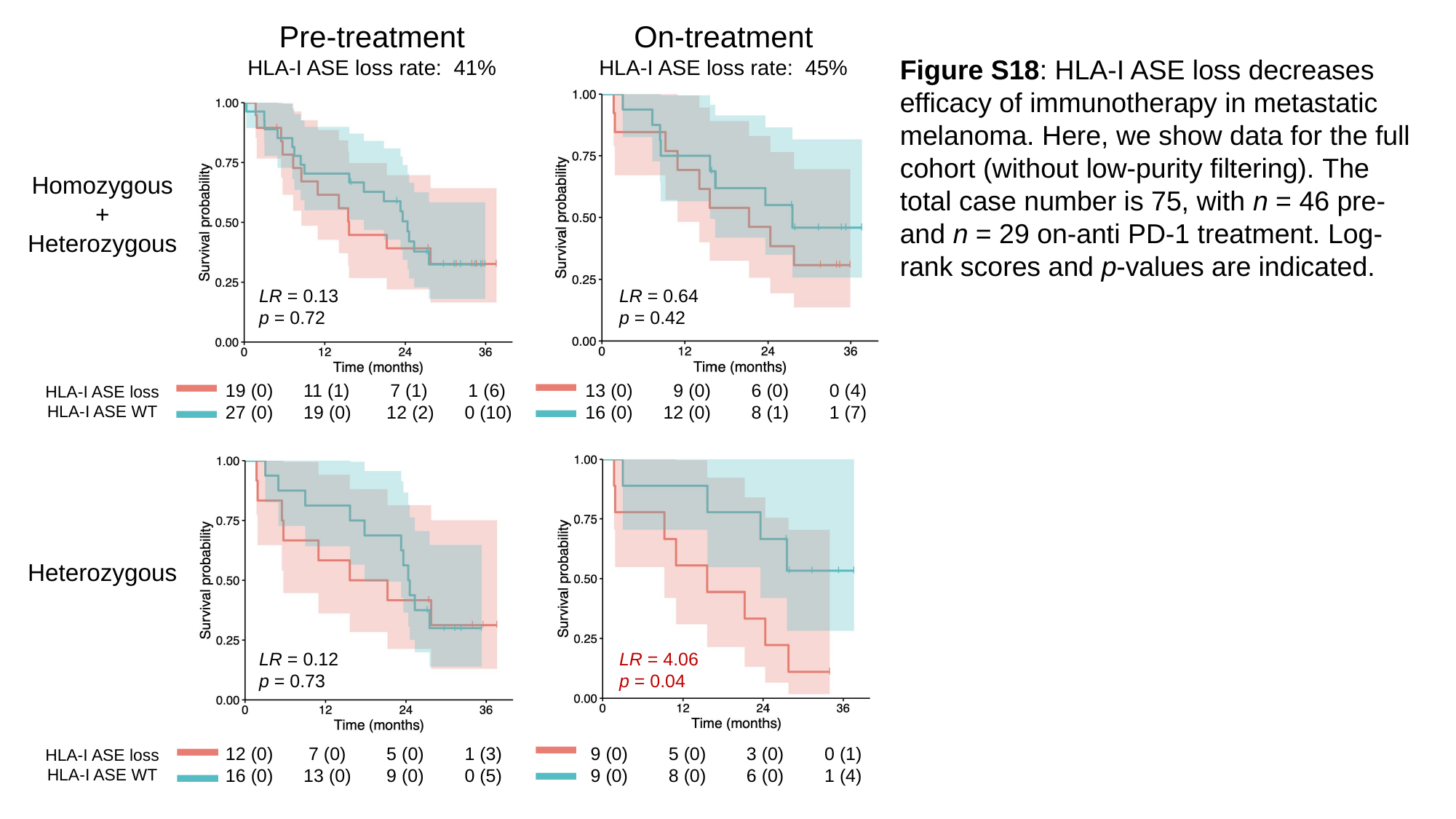

On-treatment
HLA-I ASE loss rate: 45%
Pre-treatment
HLA-I ASE loss rate: 41%
Figure S18: HLA-I ASE loss decreases efficacy of immunotherapy in metastatic melanoma. Here, we show data for the full cohort (without low-purity filtering). The total case number is 75, with n = 46 pre- and n = 29 on-anti PD-1 treatment. Log-rank scores and p-values are indicated.
Homozygous
+
Heterozygous
LR = 0.64
p = 0.42
LR = 0.13
p = 0.72
13 (0) 9 (0) 6 (0) 0 (4)
16 (0) 12 (0) 8 (1) 1 (7)
19 (0) 11 (1) 7 (1) 1 (6)
27 (0) 19 (0) 12 (2) 0 (10)
HLA-I ASE loss
HLA-I ASE WT
Heterozygous
LR = 4.06
p = 0.04
LR = 0.12
p = 0.73
9 (0) 5 (0) 3 (0) 0 (1)
9 (0) 8 (0) 6 (0) 1 (4)
12 (0) 7 (0) 5 (0) 1 (3)
16 (0) 13 (0) 9 (0) 0 (5)
HLA-I ASE loss
HLA-I ASE WT

## Slide 21
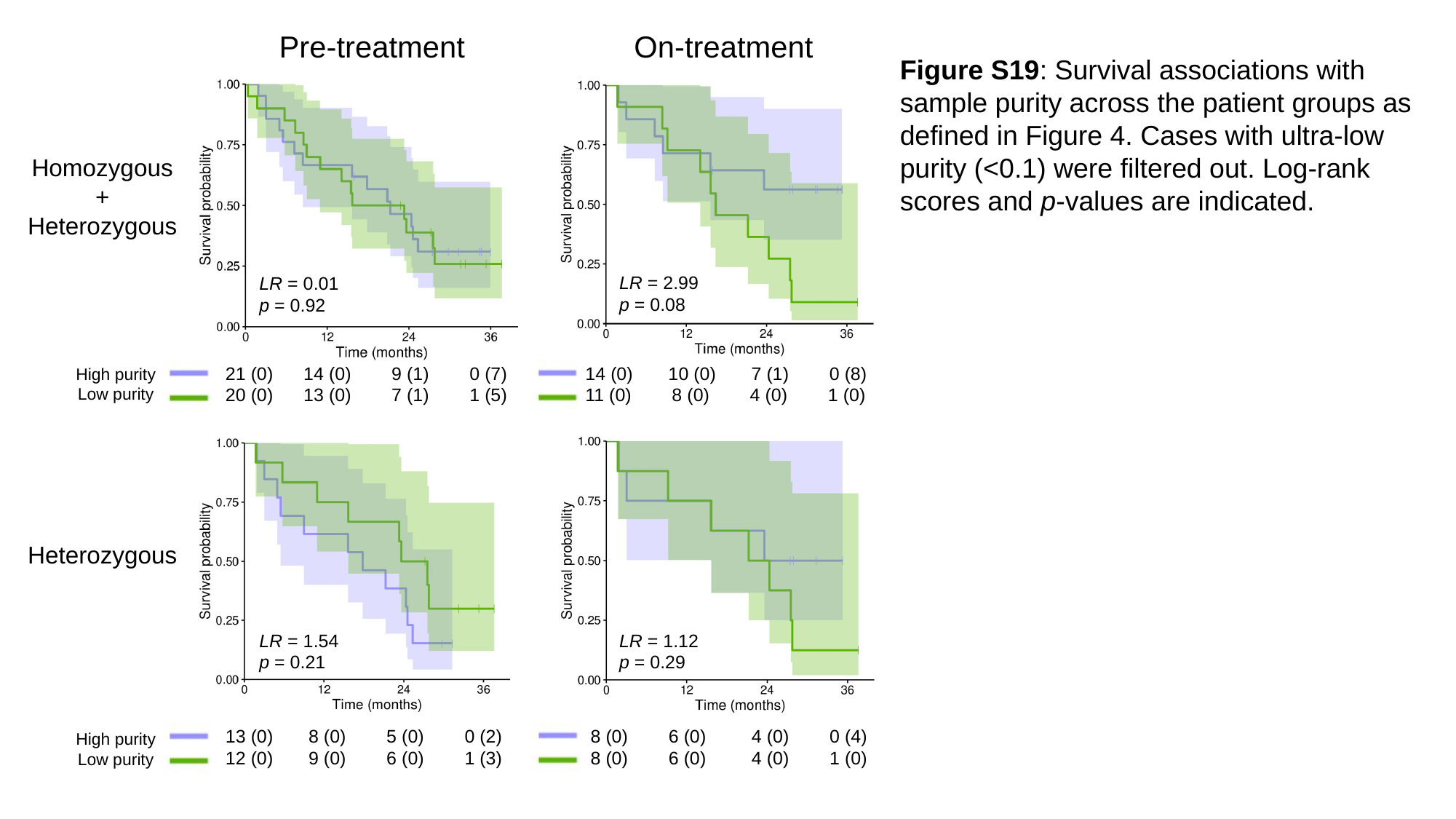

Pre-treatment
On-treatment
Figure S19: Survival associations with sample purity across the patient groups as defined in Figure 4. Cases with ultra-low purity (<0.1) were filtered out. Log-rank scores and p-values are indicated.
Homozygous
+
Heterozygous
LR = 2.99
p = 0.08
LR = 0.01
p = 0.92
14 (0) 10 (0) 7 (1) 0 (8)
11 (0) 8 (0) 4 (0) 1 (0)
21 (0) 14 (0) 9 (1) 0 (7)
20 (0) 13 (0) 7 (1) 1 (5)
High purity
Low purity
Heterozygous
LR = 1.12
p = 0.29
LR = 1.54
p = 0.21
8 (0) 6 (0) 4 (0) 0 (4)
8 (0) 6 (0) 4 (0) 1 (0)
13 (0) 8 (0) 5 (0) 0 (2)
12 (0) 9 (0) 6 (0) 1 (3)
High purity
Low purity
